# A Review of Epidemiological Research on Adverse Neurological Effects of Exposure to Ambient Air Pollution

**DOI:** 10.3389/fpubh.2016.00157

**Published:** 2016-08-05

**Authors:** Xiaohui Xu, Sandie Uyen Ha, Rakshya Basnet

**Affiliations:** ^1^Department of Epidemiology and Biostatistics, School of Public Health, Texas A&M Health Science Center, College Station, TX, USA; ^2^College of Public Health and Health Professions, University of Florida, Gainesville, FL, USA

**Keywords:** air pollution, neurotoxicity, cognitive function, particulate matter, neurological effects, brain

## Abstract

There is a growing body of epidemiological research reporting the neurological effects of ambient air pollution. We examined current evidence, identified the strengths and weaknesses of published epidemiological studies, and suggest future directions for research in this area. Studies were identified through a systematic search of online scientific databases, in addition to a manual search of the reference lists from the identified papers. Despite being a relatively new area of investigation, overall, there is mounting evidence implicating adverse effects of air pollution on neurobehavioral function in both adults and children. Further research is needed to expand our understanding of these relationships, including improvement in the accuracy of exposure assessments; focusing on specific toxicants and their relationships to specific health endpoints, such as neurodevelopmental disorders and neurodegenerative diseases; investigating the combined neurological effects of multiple air pollutants; and further exploration of genetic susceptibility for neurotoxicity of air pollution. In order to achieve these goals collaborative efforts are needed from multidisciplinary teams, including experts in toxicology, biostatistics, geographical science, epidemiology, and neurology.

## Introduction

Over the past 30 years, extensive evidence has shown that air pollution affects cardiovascular and respiratory morbidity and mortality in both adults and children across the world (1–4). Air pollution has also been consistently and widely associated with elevated risks of adverse pregnancy outcomes such low birth weight (5, 6), preterm delivery (7, 8), intrauterine growth retardation (9, 10), and birth defects (10, 11). More recently, there have been studies examining the link between air pollution and adverse neurological outcomes.

Incidence rates of diseases of the nervous system, such as neurodegenerative diseases in adults and neurodevelopmental disorders in children, have increased over the past years (12, 13). Fox et al. conducted a cumulative risk assessment for 40 ambient hazardous air pollutants (HAPs) based on either the single-effect toxicological data from the U.S Environmental Protection Agency (EPA) or their own multiple-effect toxicological database. The cumulative risk assessment of 40 HAPs revealed that neurological effects ranked the 2nd out of 17 health effects – only after respiratory effects – regardless of the data sources (14). The nervous system, particularly the central nervous system (CNS), is vulnerable to oxidative stress because it has high metabolic demands, high energy use, widespread axonal and dendritic networks, high cellular content of lipids and proteins, and low levels of endogenous scavengers, such as vitamin C and superoxide dismutase, which, to some extent, may be due to the CNS being isolated (15). The CNS in a child could be especially susceptible to oxidative stress from environmental toxicants because of its underdeveloped barrier and a wide time window of conformation. In fact, a systematic review by Landrigan et al. has found that exposure to pollution in early life has special implications in neurogenerative effects later in life (16). Thus, because of the ubiquity of toxicants present in air, potential neurological effects need to be evaluated.

Air pollution is a multifaceted environmental toxicant that comprises a diverse mixture of particulate matters (PMs), including organic components and metals, and gases, such as nitrogen oxides, sulfur oxides, and ozone. It can generate reactive oxygen species, deplete endogenous antioxidants, alter mitochondrial functions, and produce oxidative damage to lipids and DNA (17). Inflammation and oxidative stress have been recognized as the main potential mechanisms through which air pollution causes damage to cardiovascular and respiratory systems (18–20). Thus, it is logical to hypothesize that air pollution could also cause damage to the nervous system through oxidative stress pathways. Oberdorster and Utell first raised the concern that the brain may be targeted by ultrafine PM (21). Since then, several population-based studies have been conducted to evaluate the adverse neurologic effects of exposure to ambient air pollution.

The purpose of this review is to systematically examine population-based studies evaluating the relationship between air pollution and neurological outcomes to determine if there is sufficient evidence to suggest a causal link and to identify knowledge gaps to help guide new research efforts.

## Materials and Methods

We searched all publications included in the electronic databases of PubMed (from 1966 to present, National Library of Medicine, Bethesda, MD, USA), Google Scholar (Google Inc., Mountain View, CA, USA), and the Institute of Scientific Information Web of Knowledge (from 1966–present, Thompson Scientific, Philadelphia, PA, USA). We used different combinations of MeSH headings of “air pollution,” “air pollutant,” “PM,” “HAP,” or “AQS” with any of the following: “cognitive,” “cognition,” “nervous system diseases,” “neurotoxicity syndromes,” “neurodegenerative diseases,” “neurodevelopmental diseases,” “Alzheimer,” “Parkinson,” “amyotrophic lateral sclerosis (ALS),” “Huntington,” “autism,” “attention-deficit hyperactivity disorder (ADHD),” and “learning disability.” Furthermore, we also reviewed the reference lists of the identified papers and manually searched for additional publications. All research articles pertaining to studying health effects of ambient air pollution on cognitive performance, neurological symptoms, and neurobehavioral disorders were selected for a full review. In addition, we included abstracts of conference presentations if the information provided was adequate to make an evaluation. We excluded studies that were not published in English or those that were not original studies. In addition, we excluded research on stroke as the relationship between air pollution and cardiovascular diseases have been extensively studied. Because of our focus on ambient air pollution, we also excluded studies that investigated air pollutants related to occupational, accidental exposure, or indoor generated pollutants. Since neurological effects of lead exposures are well-established, we did not include studies pertaining to this topic. The search was last updated in May 2016.

## Results

**Figure 1:**
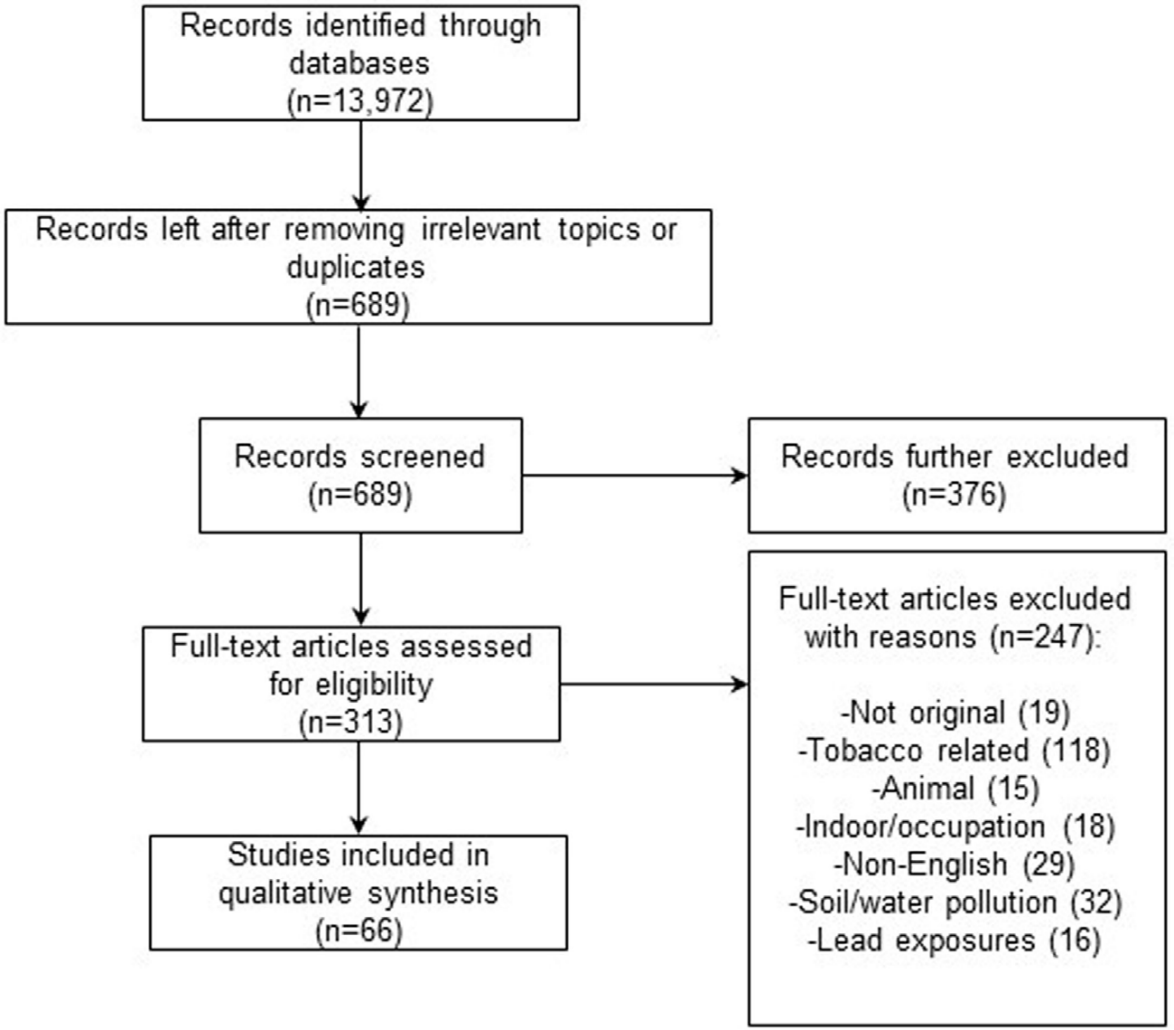
**Study selection**.

Figure 1 illustrates the study inclusion and exclusion process. Initial search from three large search engines yielded more than 13,000 entries, yielding 689 unduplicated articles. After applying inclusion/exclusion criteria, 66 studies remained in this review. Among them, one study examined both children and adult (22), and the other study examined multiple outcomes (23). Despite being a relatively new area of investigation, there is mounting evidence implicating adverse effects of air pollution on the nervous system in both adults and children. Ambient air pollution may be associated with biological changes in the brain, such as change in brain activity, increase in inflammatory reaction, and pathological changes in brain tissues in children and/or adults (22, 24, 25). Moreover, studies consistently showed that ambient air pollution, particularly traffic-related air pollution (TRAP), is associated with various adverse neurological health effects, including decreased neurocognitive abilities, such as memory and motor responses; nervous system sequelae, such as fatigues, headache, and inability to concentrate; and neurological disorders, such as Alzheimer’s, Parkinson’s, ADHD, and autism. In adults, a total of 26 studies have been identified for a full review. Among them, 4 studies on brain histology and activity (24–27); 13 studies examined the health effects of ambient or traffic air pollution on cognitive functions (28–40); 7 study examined the effects of ambient air pollutants on neurodegenerative diseases (41–47); and 1 on neurological symptoms, such as headache and fatigue and trouble concentrating (48). In children, 41 studies have been selected for a full review: 3 studied brain histological anomalies (22, 23, 49); a total of 24 studies have investigated the effects of prenatal and/or postnatal exposure to air pollution on neurodevelopment and cognitive functions among children (23, 50–72); and 15 studies investigated the effects of air pollution on risks of neurodevelopmental disorders, such as ADHD (73–75) and autism (76–87). These studies are individually summarized and discussed below and in Tables 1 and 2 for adults and children, respectively. We also critically evaluate potential epidemiological limitations regarding the published studies we reviewed in this area, including measurement of exposures, study design, confounding, and biases in the Section “Discussion.”

**Table 1:** **Overview of current evidence concerning the neurotoxicity of ambient or traffic-related air pollution in adults (26 publications)**.

Reference	Study location	Study design	Participants	Exposure	Outcome	Results	Covariates
Calderón-Garcidueñas et al. (24)	Mexico	Cross-sectional	19 deceased subjects with age ranging from 32 to 83	High level (Mexico City) vs. low levels (Polotitlán) of air pollution	Brain inflammation biomarkers: COX2 expression and Aβ42 accumulation	Residents of cities with high air pollution had significantly higher COX2 expression and astrocytic accumulation of Aβ42 in brain tissues: 1.2 × 10^2^ molec per fmole vs. 4.0 × 102 molec per fmole (*p* < 0.05) Higher COX2 expression was correlated with oxidative DNA damage measured by AP sites (*r* = 0.89, *p* = 0.001).	N/A
Inserra et al. (33)	Nebraska, USA	Cross-sectional	335 participants aged ≥16	H_2_S exposure determined by Kriging: High (≥90 bbp) vs. low (<50 bbp)	Cognitive, motor response, sensory, and mood/affect	Marginally poorer performance on a test of memory (OR = 0.6, 95% CI: 0.4, 1.0) and a test of grip strength (OR = 0.7, 95% CI: 0.4, 1.0) in highly H_2_S-exposed residents No significant differences were observed for other constructs	Age, sex, and testing language
Finkelstein and Jerrett (43)	Toronto and Hamilton, Canada	Retrospective cohort	110,348 adult participants (age not reported)	NO_2_ levels and Manganese (Mn) fraction determined by spatial interpolation	Parkinson’s disease	In Hamilton: – Mn fraction: OR = 1.034 (1.00–1.07, *p* = 0.049) per 10 μg/m^3^ increase in Mn in TSP. – NO_2_: OR = 1.055 (1.00, 1.11, *p* = 0.045) per 1-ppb increase. In Toronto: – no significant findings	Year of birth, sex
Apte et al. (48)	USA	Cross-sectional	4,200 respondents (age range not reported)	Ozone determined by nearest station	Building-related symptoms: fatigue or trouble concentrating, and headache	Fatigue or trouble concentrating: OR = 1.03, 95% CI: 1.01, 1.05, per 10 μg/m^3^ increase of late workday ozone Headache: OR = 1.03, 95% CI: 1.00, 1.05, per 10 μg/m^3^ increases of late workday ozone	Season, sex age, smoking status, environmental sensitivities, relative humidity, and thermal exposure
Cruts et al. (25)	UK	Double-blinded randomized crossover	10 human volunteers, aged 18–39	Diesel exhaust produced by an engine	Brain activity measured by quantitative electroencephalography	Increase in median power frequency in the frontal cortex in response to exposure to diesel exhaust for 30 min	N/A
Chen and Schwartz (31)	USA	Ecological	1,764 adult participants (aged 37.5 ± 10.9 years)	Annual PM_10_ and ozone level at the county of residence determined by inverse distance weighting	Cognitive functions and reaction time	Per 100 ppb increase in ozone: – SDST score increases: 0.12 (95% CI: 0.01, 0.23) – SDLT score increases: 0.57 (95% CI: 0.08, 1.06) (Increasing score means increasing reaction time) No significant findings for PM_10_	Age, sex, race, education, employment status, smoking status, alcohol consumption, family size, annual family income, poverty-income ratio, and type of residence (urban/rural), CVD risk factors
Ranft et al. (36)	Ruhr district, Germany	Cohort	399 women aged 68–79 years	Distance to busy streets	Cognitive functions	Living near busy streets within 50 m was associated with mild cognitive impairment: CERAD-Plus test: −3.8 (95% CI: −7.8, 0.1); stroop test: −5.1 (95% CI: −8.2, −2.0); sniffing test: −1.3 (95% CI: −2.4, −0.2).	Age, years live in area, smoking status, environmental quality, indoor pollution, history of chronic diseases, depression, education
Calderón-Garcidueñas et al. (22)	Mexico	Cross-sectional	44 adults and children aged 2–40 years	High (Mexico City)/low (Polotitlan) levels of air pollution	The University of Pennsylvania Smell Identification Test (UPSIT) score Olfactory bulbs (OB) pathology	MC subjects had significantly lower UPSIT scores: 34.24 ± 0.42 vs. controls 35.7 ± 0.40, *p* = 0.03 The exposed groups exhibited remarkable immunoreactivity to Aβ42 and α-synuclein, and OB endothelial hyperplasia, and neuronal accumulation of particles	N/A
Power et al. (35)	USA	Retrospective cohort	680 men (mean ± SD, 71 ± 7 years of age)	Black carbon determined by land-use regression models	Cognitive functions	Low-mini-mental status examination score (<25): OR = 1.3 (1.1–1.6) for each doubling in black carbon concentration Each doubling in black carbon concentration was also associated with a 0.054 SD lower cognitive test score (95% CI: −0.103, −0.006)	Age, education, first language, computer experience, physical activity, alcohol consumption, diabetes, dark fish consumption, percentage of residential census tract that is non-white, percentage of residential census tract adults with a college degree, indicator for first cognitive assessment, and indicator for part-time resident
Wellenius et al. (39)	USA	Retrospective cohort	765 adults seniors aged ≥65 years	Distance to major road way	Cognitive functions	An interquartile range decrease (851.2 m) in residential distance to major roadway is associated with decreased scores for immediate [−0.6 (−1.1 to −0.1)] and delayed [−0.4 (−0.7 to −0.1)] recall, letter [−1.4 (−2.7 to −0.2)] and category influency [−0.7 (−1.1 to −0.3)], and trail making test scores [10.5 (4.0–17.1)]	Age, sex, race, history of stroke, history of smoking, education, visit number, body mass index, physical activity, household income, percentage of neighborhood population that is non-white, and percentage of neighborhood population with a college degree or above
Weuve et al. (40)	USA	Cohort	19,409 US women aged 70–81 years	PM_2.5–10_, and PM_2.5_ determined by USEPA spatiotemporal smoothing models	Global cognitive scores measured by Telephone Interview for Cognitive Status (TICS)	For long-term exposure (7–14 years): PM_2.5–10_ exposure: – The 2-year change in standardized cognitive scores for each 10 μg/m^3^ increment in PM_2.5–10_ exposure is: −0.020, 95% CI: −0.032, −0.008 PM_2.5_ exposure: – The 2-year change in standardized cognitive scores for each 10 μg/m_3_ increment in PM_2.5_ exposure is: −0.018 (95% CI: −0.035, −0.002)	Age, education, husband’s education, long-term physical activity, and long-term alcohol consumption
Calderón-Garcidueñas et al. (26)	Mexico	Cross-sectional	59 participants (age 33.06 ± 4.8 years)	Air pollution levels High: Mexico city Low: Tlaxcala and Veracruz	Brain inflammation markers: COX2 and IL1β	COX2 in the frontal cortex was significantly elevated in Mexico City subjects vs. control, *p* = 0.008. Exposed subjects also had higher IL1β expression in the frontal cortex, *p* = 0.0002. COX2 in olfactory bulb was significantly elevated vs. control, *p* = 0.005	N/A
Loop et al. (34)	USA	Cohort	20,150 men and women aged ~64 ± 9.2 years	Satellite-derived estimate of 1-year mean PM_2.5_ concentration	Cognitive status assessed over the telephone using the Six-Item Screener (SIS)	A 10-mg/m^3^ increase in PM_2.5_ concentration was not reliably associated with an increased odd of incident impairment (OR = 1.26 95% CI: 0.97, 1.64).	Age, sex, race, region, income, education level, depressive symptoms, smoking status, alcohol use, level of exercise, temperature, season, incident stroke, length of follow-up
Ailshire and Crimmins (29)	USA	Cross-sectional	13,996 men and women aged 50 years or older	Average PM_2.5_ concentration from each monitoring station were weighted using the inverse of the distance	Cognitive function based on the Telephone Interview for Cognitive Status	Older adults living in areas with higher PM_2.5_ concentrations (highest quartile) had worse cognitive function (β = −0.26, 95% CI: −0.47, −0.05).	Age, sex, and race/ethnicity, educational attainment, current employment status, income, smoking behavior, census tract-level proportion of residents aged 25 years or older without a high school degree and median household income
Chang et al. (42)	Taiwan	Retrospective cohort	29,547 adults ages ≥50	Yearly average NO_2_ and CO concentrations measured at the monitoring stations	Dementia assessed using International Classification of Disease, Ninth Revision, Clinical Modification (ICD-9-CM)	The adjusted hazard ratios (HRs) of dementia for all participants in Q2, Q3, and Q4 compared to Q1 were as follows: for NO_2_: 1.10 (95% CI: 0.96, 1.26), 1.01 (95% CI: 0.87, 1.17), and 1.54 (95% CI, 1.34, 1.77); and for CO: 1.07 (95% CI: 0.92, 1.25), 1.37 (95% CI: 1.19, 1.58), and 1.61 (95% CI: 1.39, 1.85)	Age, sex, monthly income, diabetes, hypertension, chronic obstructive pulmonary disease, alcoholism, and urbanization
Gatto et al. (32)	Los Angeles, California, USA	Cross-sectional	1,496 adults ages 60.5 ± 8.1 years	Average O_3_, PM_2.5_, and NO_2_ concentration from 2000–2006 estimated using monitoring data and inverse distance weighing	Global cognition and six domains of cognitive function using a battery of tests	Increasing exposure to PM_2.5_ was associated with lower verbal learning (β = −0.32 per 10 μg/m^3^; 95% CI: −0.63, 0.00) NO2 >20 ppb was associated with lower logical memory [β = −0.62 (−1.35, 0.11)] O3 exposure above 49 ppb was associated with lower executive function [β = −0.66 (−1.35, 0.03)]	Age, gender, race, education, income, study, and mood
Heydarpour et al. (44)	Tehran, Iran	Cross-sectional (cluster analysis)	2,188 multiple sclerosis among 8.2 million background population	PM_10_, SO_2_, NO, NO, NO_x_ concentration during the year 2010 estimated using LUR	Multiple sclerosis (MS) diagnosis meeting McDonald’s criteria	Prevalent MS cases had a clustered pattern in Tehran. A significant difference in exposure to PM_10_, SO_2_, NO_2_, and NOx (*p* < 0.001) was observed in MS cases compared with controls	N/A
Tonne et al. (37)	London, England	Cohort	2867 adults ages 66 ± 6 years	Average PM_10_ and PM_2.5_ concentration estimated by dispersion model at home address	Decline in reasoning, memory, and phonemic and semantic fluency	Higher PM_2.5_ of 1.1 μg/m^3^ (lag 4) was associated with a 0.03 (95% CI: −0.06 to 0.002) 5-year decline in standardized memory score and a 0.04 (−0.07 to −0.01) decline when restricted to participants remaining in London between study waves	Age, sex, ethnicity, marital status, educational achievement, socioeconomic position, smoking status, alcohol use, frequency of fruit and vegetable consumption, physical activity, blood pressure, serum cholesterol levels, prevalence of stroke, coronary heart disease and diabetes, and frequency of depressive symptoms
Ailshire and Clarke (28)	USA	Cohort	780 men and women aged 55 and older	Annual PM_2.5_ levels based on air monitoring data	Cognitive function assessed with an abbreviated form of the Short Portable Mental Status Questionnaire (SPMSQ)	Those living in areas with greater exposure to PM_2.5_ had an error rate 1.5 times greater than those exposed to lower PM_2.5_ concentrations (IRR = 1.53; 95% CI: 1.02, 2.30 for 10 μg/m^3^ increment)	Age, gender, race, education, income, marital status, employment status, and residential tenure
Bowler et al. (30)	Ohio, USA	Cross-sectional	186 adults 56 ± 10.8 years	Air borne manganese (Mn) estimated using U.S. EPA’s AERMOD dispersion model	Cognitive function(s) assessed by a battery of tests	Significant relationships (*p* < 0.05) were found between Mn exposure and performance on working and visuospatial memory (e.g., Rey-O Immediate β = −0.19, Rey-O Delayed β = −0.16) and verbal skills (e.g., similarities β = −0.19)	Education, town of residence, age, income, ethnicity, years of residence, BMI, employment status, sex, race
Jung et al. (45)	Taiwan	Cohort	95,690 individuals ages ≥65	O_3_ and PM_2_*_._*_5_ exposure estimated using inverse distance weighting	Newly diagnosed Alzheimer’s disease assessed by ICD-9-CM	The adjusted HR for AD was weakly associated with a raised concentration in O3 at baseline per increase of 9.63 ppb (adjusted HR: 1.06). There was a 211% increase AD risk per increase of 10.91 ppb in O_3_ over the follow-up period (95% CI: 2.92, 3.33), and a 138% increase AD risk per increase of 4.34 μg/m^3^ in PM_2.5_ over the follow-up period (95% CI: 2.21, 2.56)	Age, gender, income, and other comorbidities (e.g., diabetes, hypertension, myocardial infarction, angina pectoris, stroke, peripheral artery disease, asthma, COPD)
Kalkbrenner et al. (83)	North Carolina and California, USA	Case–control	979 cases and 14,666 controls	PM_10_ exposure	Autism Spectrum Disorder	PM_10_ exposure at the third trimester was associated with autism (OR = 1.36, 95% CI: 1.13–1.63)	Year, state, maternal education and age, race/ethnicity, neighborhood-level urbanization and median household income, and seasonality
Malek et al. (46)	Pennsylvania, USA	Case–control	51 cases and 51 matched controls ages’ ≥18 (matched on age of onset, race, sex)	Hazardous air pollutants (HAPs) exposures on census-tract level estimated by U.S. Environmental Protection Agency (EPA) National-Scale Air Toxics Assessment (NATA)	Amyotrophic lateral sclerosis (ALS)	Residential exposure to aromatic solvents significantly elevated the risk of ALS among cases compared to controls in 2002 (OR ¼ 5.03, 95% CI: 1.29–19.53) and 1999 (OR ¼ 4.27, 95% CI: 1.09–16.79)	Education, smoking, and other exposure groups (metals, pesticides, other HAPs)
Angelici et al. (41)	Lombardy region, Italy	Cohort	8287 hospital admissions (linked with MS) in 107 hospitals between 2001–2009	Exposure to particulate matter	Multiple sclerosis hospital admission	A higher RR of hospital admission for MS relapse was associated with exposure to PM_10_ at different time intervals. Hospital admission for MS increased 42% (95% CI: 1.39–1.45) on the days preceded by 1 week with PM10 levels in the highest quartile. The *p*-value for trend across quartiles was <0.001	Number of MS hospital admissions, PM_10_ concentration, day of the week on the day of admission, seasonality, day-off, resident population in each area, smoothing spline functions of time, day of the week and smoothing splines of 3-day lags of average temperature with 3 df
Cliff et al. (27)	Vancouver, Canada	Controlled blinded crossover	27 healthy adults aged 19–49 years	Exposure to air pollution	Biomarkers of systemic and CNS inflammation	At no time-point following exposure to DE was a significant increase in concentration from baseline seen for IL-6, TNF-a, S100b, or NSE relative to FA exposure. Similarly, no significant decrease in BDNF concentration from baseline was seen following DE exposure, relative to FA. Furthermore, the repeated measures ANOVA considered for all time-points and biomarkers revealed no significant time-exposure interaction	
Oudin et al. (47)	Northern Sweden	Longitudinal	1806 participants from the Betula study	Long-term exposure to ambient (traffic-related) air pollution	Dementia incidence	Participants with the highest exposure were more likely than those with the lowest exposure to be diagnosed with dementia (Alzheimer’s disease or vascular dementia), with a hazard ratio (HR) of 1.43 (95% CI: 0.998–2.05 for the highest vs. the lowest quartile). The estimates were similar for Alzheimer’s disease (HR 1.38) and vascular dementia (HR 1.47). The HR for dementia associated with the third quartile vs. the lowest quartile was 1.48 (95% CI: 1.03–2.11)	Education, physical activity, smoking, sex, BMI, waist-hip ratio (WHR), alcohol, age, and apolipoprotein E (ApoE4)
Tzivian et al. (38)	Three adjacent cities (Bochum, Essen and Mülheim/Ruhr) in the highly urbanized German Ruhr Area	Cross-sectional	4814 participants aged 45–75 years from a population-based cohort study	Long-term air pollution and traffic noise exposures	Mild cognitive impairment in older adults	Long-term exposures to air pollution and traffic noise were positively associated with MCI, mainly with amnestic subtype. An interquartile range increase in PM_2.5_ and a 10 dB (A) increase in L_DEN_ were associated with overall MCI (OR: 1.16, 95% CI: 1.05–1.27) and (1.40, 95% CI: 1.0–1.91), respectively, and with aMCI (1.22, 96% CI: 1.08–1.38) and (1.53, 95% CI: 1.05–2.24), respectively. In two-exposure models, AP and noise associations were attenuated [i.g., for aMCI: PM_2.5_ 1.13 (0.98, 1.30) and L_DEN_ 1.46 (1.11, 1.92)]	Age, sex, socioeconomic status, alcohol consumption in drinks per week, smoking status, environmental tobacco smoke, any regular physical activity, t, BMI, history of coronary heart disease, low-density lipoprotein, type 2 diabetes mellitus, use of statins and anti-hypertensive medication during 7 days before examination and symptoms of depression

**Table 2:** **Overview of current evidence concerning the neurotoxicity of ambient or traffic-related air pollution in children (41 publications)**.

Reference	Study location	Study design	Participants	Exposure	Outcomes	Results	Covariates
Perera et al. (69)	New York City, USA	Prospective cohort study	183 children 3 years of age	Prenatal exposure to PAHs (individual monitoring at the 3rd trimester)	Neurodevelopment in first 3 years of life: – Bayley Scale of Infant Development. – Child Behavior Checklist	Mental development index at age 3: −5.69 (95% CI: −9.05 to −2.33); cognitive developmental delay: OR = 2.89 (95% CI: 1.33–6.25) in the highest vs. the lowest category of PAH exposure	Prenatal exposure to ETS and chlorpyrifos, and maternal IQ and education
Windham et al. (76)	San Francisco, USA	Case–control	284 children with ASD diagnosed before 9 years and 657 controls	Hazardous air pollutants (HAPs) levels at census tract level estimated by EPA Gaussian Dispersion Models	Autistic spectrum disorder	Chemical chlorinated solvents – Methylene chloride: OR = 1.50 (1.06–2.13) third quartile – Trichloroethylene: OR = 1.47 (1.03–2.08) forth quartile – Vinyl chloride: OR = 1.75 (1.25–2.43) forth quartile Metals – Cadmium: OR = 1.54 (1.08–2.20) forth quartile – Mercury: OR = 1.92 (1.36–2.71) forth quartile – Nickel: OR = 1.46 (1.04–2.06) forth quartile	Maternal age, education, and child race
Calderón-Garcidueñas et al. (23)	Mexico	Cross-sectional Descriptive	73 children aged 10.5 ± 3.7 and 9.3 ± 3.7 years in two locations	High (Mexico City)/low (Polotitlan) levels of air pollution	Cognitive deficit: WISC-R Brain abnormalities: MRI	Highly exposed children had: – Poorer performance in the digit span (*p* = 0.014), information (*p* = 0.019), and arithmetic (*p* = 0.031) – Higher proportion of prefrontal white matter hyperintense lesions than low exposed groups.		Age, gender
Suglia et al. (58)	Boston, MA	Prospective cohort study	202 children (ages 9.7 ± 1.7 years)	Black carbon estimated by land-use regression model	Cognitive functions: Kaufman Brief Intelligence Test (KBIT); The Wide Range Assessment of Memory and Learning (WRAML)	Per interquartile range increasing in log black carbon: KBIT: matrices score: −4.0 (−7.6 to −0.5), composite score: −3.4 (−6.6 to −0.3); WRAML: visual score: −5.4 (−8.9 to −1.9), general score: −3.9 (−7.5 to 0.3)	Age, gender, primary language, mother’s education, passive smoking, birth weight and blood lead level.
Tang et al. (71)	Chongqing, China	Prospective cohort study	110 children under 2 years of age	PAH–DNA adducts level in cord blood	Developmental quotients (DQ) in motor, adaptive, language and social areas at age of 2 years old	Per unit of cord adducts increase: Motor area DQ: −16.01 (−31.30, −0.72); Average DQ: −14.57 (−28.77, −0.38); OR for motor developmental delay is 1.91 (1.22, 2.97) for every 0.1 unit increase in cord adduct	Cord lead level, environmental tobacco smoke, sex, gestational age, and maternal education
Wang et al. (61)	Quanzhou, China	Cross-sectional	861 children ages 8–10 years	Traffic-related air pollution: low vs. exposed school based on NO_2_ and PM_10_ levels (ecologically determined by air monitors and passive samplers)	Neurobehavioral functions: cognitive, motor, sensory and psychomotor.	Compared high to low exposure schools, odds of lower performance: visual response speed: visual simple reaction time-preferred hand test (OR = 1.67, *p* = 0.044), and the visual simple reaction time-non-preferred hand test (OR = 1.83, *p* = 0.017); Speed and attention, measured by the continuous performance test (OR = 2.40, *p* = 0.001); Accuracy and speed, measured by the digit symbol test (OR = 1.38, *P* = 0.019); Psychomotor stability, measured by the pursuit aiming test (OR = 1.61, *P* < 0.001); and motor coordination, measured by the sign register test (OR = 1.94, *P* < 0.001)	Age, BMI, educational attainment of father, birth weight, delivery method, passive smoking, open kitchen, familiarity with computer games, household fuel, breast feeding
Calderón-Garcidueñas et al. (22)	Mexico	Cross-sectional	44 adults and children aged 2–40 years	High (Mexico City)/low (Polotitlan) levels of air pollution	The University of Pennsylvania Smell Identification Test (UPSIT) score; Olfactory bulbs (OB) pathology	MC subjects had significantly lower UPSIT scores: 34.24 ± 0.42 vs. controls 35.7 ± 0.40, *p* = 0.03 The exposed groups exhibited remarkable immunoreactivity to Aβ42 and α-synuclein, and OB endothelial hyperplasia, and neuronal accumulation of particles	N/A
Freire et al. (53)	Spain	Cohort	210 children ages 4–5 years	NO_2_ exposure estimated by land-use regression models	Children’s motor and cognitive abilities measured by McCarthy Scales of Children’s Abilities (MSCA)	For children exposed to higher NO_2_ (>24.75 mg/m^3^): general cognitive score: decrease of 4.19 points; quantitative memory: decreases of 6.71 points; working memory: decrease of 7.37 points; gross motor: decrease of 8.61 points	Birth weight, birth length, gestational age, smoking during pregnancy, place of residence, maternal and paternal educational level, maternal occupational status at child age of 4, maternal marital status, mother’s parity at childbirth, breastfeeding (weeks), mother-to-infant attachment score, and maternal mental health score
Kalkbrenner et al. (77)	North Carolina and West Virginia, USA	Case–control	3212 children ≤8 years of age	Prenatal exposure to census-tract HAPS level estimated by 1996 National Air Toxics Assessment annual-average model	Autism spectrum disorder at age 8	Comparing 80th percentile vs. 20th percentile of prenatal exposure: quinoline (OR = 1.4, 95% CI: 1.0–2.2); Styrene (OR = 1.8, 95% CI: 1.0–3.1)	Race, maternal education, maternal age, smoking in pregnancy, marital status, census tract median household income, and urbanicity
Calderón-Garcidueñas et al. (50)	Mexico	Cross-sectional	30 children with ages ~7.0 ± 0.7 years	High (Mexico City)/low (Polotitlan) levels of air pollution	Cognitive functions	Highly exposed healthy children had exhibited selective impairment in IQ subscales tapping on attention, short-term memory and learning abilities	Year, brain histology
Guxens et al. (64)	Spain	Prospective cohort study	1,889 children ~14 months	Personal passive samplers and land regression models level of prenatal exposures to NO_2_ and benzene	Cognitive functions: Bayley Scales of Infant Development	Exposure to NO2 and benzene are inversely associated with mental development (−4.13 (−7.06, −1.21) and −4.37 (−6.89, −1.86) for NO2 and benzene, respectively) in the group of low intakes of fruits/vegetables during pregnancy	Psychologist, child’s sex and age, maternal education, age, alcohol use, height, pre-pregnancy BMI, and dietary, etc.
Siddique et al. (73)	Delhi, India	Cross-sectional	1819 children 2–17 years	PM_10_ estimated by surrounding monitoring stations	ADHD	<120 μg/m3: 1.00 (referent); 120–139 μg/m3: 1.824 (1.070–3.629); 140–200 μg/m3: 2.201 (1.162–5.032); >200 μg/m3: 2.770 (1.381–5.555)	Age, gender, socioeconomic status, and parental smoking, indoor pollution
Volk et al. (78)	California, USA	Case–control	563 children between 24 and 60 months	Proximity to freeways	Autism	Mothers living > 309m from freeways: OR = 1.86; 95% CI: 1.04–3.45. Proximity to a freeway during the third trimester: OR = 2.22; 95% CI: 1.16–4.42	Child sex, child race/ethnicity, maximum education of parents, maternal, and maternal smoking during pregnancy
Clark et al. (51)	United Kingdom	Cross-sectional	719 children who were 9–10 years	SO_2_ determined by emission dispersion and regression models	Cognitive functions; working and episodic memory	No significant findings for association between SO_2_ and cognitive functions measured by the Child Memory Scale, and The Search and Memory Task	Age, gender, mother’s educational level, parental employment status, crowding in the home, home ownership, long-standing illness, main language spoken at home, parental support for school work, and classroom window glazing, traffic noise
van Kempen et al. (60)	Netherlands	Cross-sectional	553 children (age 9–11 years)	Yearly average concentration of NO_2_ and PM_10_ estimated by land-use regression model	Cognitive performance using the Neurobehavioral Evaluation System (NES)	Air pollution at school was found to be associated with a decrease in the span length measured during Digit Memory Span Test (−0.16 (−0.28, −0.04) for each 10 mg/m^3^ increase in NO_2_)	Age, gender, employment status, crowding, home ownership, mother’s education, long-standing illness, main language spoken at home, parental support, and type of window glazing at school
Becerra et al. (79)	Los Angeles, California	Case–control	7,594 cases and 75,635 controls 3–14 years of age	CO. NO_2_, NO, O_3_, PM_2.5_, PM10 from nearest monitoring stations and LUR models	Autistic disorders diagnosed between 36 and 71 months of age	For an IQR increase, there was 12–15% increase in odds of autism for O_3_ and PM_2.5_; and 3–9% relative increases in odds for LUR-based NO and NO_2_ exposure estimates. Associations were stronger among children of mothers <high school education	Maternal age, maternal place of birth, race/ethnicity, education, type of birth, parity, insurance type, and gestational age
Jung et al. (45)	Taiwan	Cohort	49,073 children age less than 3 years	Exposures to O_3_, CO, NO2, SO_2_, PM_10_ during preceding 1–4 years estimated using inverse distance weighting	Autism spectrum disorder	Previous year exposure was associated with: 59% risk increase per 10 ppb increase in O_3_; 37% risk increase per 10 ppb in CO; 340% risk increase per 10 ppb increase in NO_2_; 17% risk increase per 1 ppb in SO_2_	Gender, municipal-level socioeconomic status (SES) of parents, and comorbidities
Newman et al. (74)	Cincinnati, Ohio, USA	Cohort	576 children ages 6.9 ± 0.3 years	Elemental carbon attributed to traffic (ECAT) during first year of life estimated by LUR	ADHD symptoms at 7 years of age assessed by the Behavioral Assessment System for Children, 2nd Edition	Highest tertile of ECAT during the child’s first year of life was significantly associated with Hyperactivity *T*-scores in the “at risk” range at 7 years of age (adjusted odds ratio (aOR) = 1.7; 95% CI: 1.0–2.7). Stronger association in children whose mothers had higher education	Environmental tobacco smoke during pregnancy and first year of life, age of home, Maternal and paternal education, household income, insurance status race/ethnicity, duration of breastfeeding, and childcare attendance
Roberts et al. (81)	14 U.S. states	Case–control	325 cases, 22,101 controls ages	U.S. Environmental Protection Agency–modeled levels of hazardous air pollutants at the time and place of birth	Autism spectrum disorder diagnosis by telephone administration of the Autism Diagnostic Interview–Revised	Perinatal exposures to the highest vs. lowest quintile of diesel, lead, manganese, and cadmium, and an overall measure of metals were significantly associated with ASD with odds ratios ranging from 1.5 (for cadmium) to 2.0 (for diesel). Associations were stronger for boys than for girls	Census tract median income and percent of residents with a college education, mother’s parents’ education, index child’s current family income, educational attainment of the mother’s partner, smoking, year of birth, maternal age at birth, and air pollution prediction model year
Volk et al. (78)	California, USA	Case–control	279 cases and 245 controls ages of 24 and 60 months	Traffic-related pollution exposure for each trimester of pregnancy and first year of life estimated by dispersion models and inverse distance weighting	Autism diagnosed by Autism Diagnostic Observation Schedules (ADOS) and Autism Diagnostic Interview-Revised (ADI-R)	Children with highest levels of modeled TRP during first year of life, and all trimesters of pregnancy were significantly more likely to have autism compared to children with the lowest exposure. Individual pollutants including PM_2.5_, PM_10_, and NO_2_ were also significantly associated at all windows	Gender, ethnicity, maximum education level of the parents, maternal age, maternal smoking during pregnancy, population density
Gong et al. (75)	Stockholm, Sweden	Cohort	3,426 twins up to 12 years of age	Residence time-weighted concentration of PM_10_ and NO_x_ during pregnancy, the first year, and the ninth year of life using dispersion modeling	Autism spectrum disorders (ASD) and attention-deficit hyperactivity disorder (ADHD) at 9 or 12 years old using DSM-IV criteria	No clear or consistent associations were found between air pollution exposure during any of the three time windows and any of the neurodevelopmental outcomes.	Gender, parity, gestational age, birthweight, maternal age at birth, maternal smoking during pregnancy, maternal marital status, parental education, family disposable income during pregnancy, child’s first year of life, and the ninth year of life, and neighborhood deprivation index
Guxens et al. (65)	Six European countries	Cohort	9482 children aged 1–6 years	NO_2_, NO_x_, PM_2.5_ absorbance, PM_10_, PM_2.5_, PM_coarse_ concentrations birth home addresses were estimated by land-use regression models	Cognitive and motor development evaluated by parents or psychologist	NO_2_ was associated with reduced psychomotor development (global psychomotor development score decreased by 0.68 points (95% CI: −1.25 to −0.11) per increase of 10 μg/m^3^)	Maternal age at delivery, educational level, country of birth, smoking during pregnancy, parity, maternal height and pre-pregnancy weight, child’s sex and date of birth
Kim et al. (67)	South Korea	Cohort	520 mother–child pairs (children ≤24 months).	PM_10_ and NO_2_ during entire pregnancy estimated by inverse distance weighting	Neurodevelopment during the first 24 months, assessed by Korean Bayley Scale of Infant Development II (K-BSID-II)	There were negative associations between maternal exposure to PM_10_ and mental developmental index (β = −2.83; *p* = 0.003) and psychomotor developmental index (β = −3.00; *p* = 0.002). NO_2_ exposure was related with impairment of psychomotor development (β = −1.30; *p* = 0.05)	Maternal age, maternal education, family income, birthweight, sex, and gestational age
Lin et al. (57)	Taiwan	Cohort	533 mother-infant pairs (children ≤18 months)	PM_10_, CO, SO_2_, NO_2_, O_3_ and hydrocarbons measured at air quality monitoring stations	Neurobehavioral development when the children were 0–18 months reported by parents	Exposures to SO_2_ during first trimester (β = −0.083, SE = 0.030), 2nd and 3rd trimester (β = −0.114, SE = 0.045), and birth 12 months (β = −0.091, SE = 0.034) were associated with decreased fine motor scores. Non-methane hydrocarbon during 2^nd^ and 3^rd^ trimester was also associated with decreased gross motor: (β = −8.742, SE = 3.512)	Maternal education, maternal education, environmental tobacco smoke exposure, infant gestational age, breastfeeding, and parental nursery type
Lovasi et al. (68)	New York, USA	Cohort	326 mother-infant pairs	Polycyclic aromatic hydrocarbon (PAH) measured by personal monitoring at a single point during third trimester	Cognitive scores at age 5 based on the Wechsler Preschool and Primary Scale of Intelligence-Revised (WPPSI-R)	Prenatal PAH exposure above the median predicted 3.5-point lower total WPPSI-R scores and 3.9-point lower verbal scores	Sex, ethnicity, maternal education, maternal IQ score, environmental tobacco smoke exposure in the home, quality of the caretaking environment, and household English language exposure.
Cowell et al. (63)	Boston, Massachusetts, USA	Cohort	258 mother-child dyads	Black carbon estimated using LUR	Cognitive scores measured by Wide Range Assessment of Memory and Learning (WRAML2) at age 6	The main effect for black carbon was not significant for any WRAML2 index; however, in stratified analyses, among boys with high exposure to prenatal stress, Attention Concentration Index scores were on average 9.5 points lower for those with high compared to low prenatal black carbon exposure	Maternal age, race/ethnicity, education, smoking status, and birthweight
Harris et al. (55)	Massachusetts, USA	Cohort	1,109 mother–child pairs	Black carbon (BC) and PM_2.5_ exposures (LUR), and residential proximity to major roadways, and near-residence traffic density during late pregnancy and childhood	Verbal and non-verbal intelligence, visual motor abilities, and visual memory at age 8	Compared with children living ≥200 m from a major roadway at birth, those living <50 m away had lower non-verbal IQ (−7.5 points; 95% CI: −13.1, −1.9), and somewhat lower verbal IQ (−3.8 points; 95% CI: −8.2 to 0.6) and visual motor abilities (−5.3 points; 95% CI: −11.0 to 0.4).	Sex, age at cognitive testing, breastfeeding duration, maternal IQ, parity, age at enrollment, marital status, education, race/ethnicity, smoking status, second hand smoke during pregnancy, father education, household ownership of gas stove, annual income, and neighborhood income
Jedrychowski et al. (66)	Krakow, Poland	Cohort	170 children	Polycyclic aromatic hydrocarbons (PAHs) exposures indicated by PAH–DNA adducts in cord blood	Cognitive function at age 7 assessed by at the Wechsler Intelligence Scale for Children-Revised (WISC-R)	Relative risk of depressed verbal IQ index increased threefold with an ln-unit increase in cord blood adducts (relative risk (RR) = 3.0, 95% CI: 1.3–6.8)	Postnatal indoor PAH exposure, maternal education, child’s gender, parity, breastfeeding practice, and prenatal and postnatal ETS
Kicinski et al.(56)	Belgium	Cohort	606 adolescents aged 14.9 ± 0.7 years	Biomarker of benzene and the amount of contact with traffic using inverse distance weighting	Neurobehavioral function assessed using the Neurobehavioral Evaluation System computerized battery of tests (NES3)	One SD increase in traffic exposure was associated with a 0.26 SD decrease in sustained attention (95% credible interval: −0.02 to −0.51)	Gender, age, smoking, passive smoking, level of education of the mother, socioeconomic status, time of the day, and day of the week
Kalkbrenner et al. (83)	USA	Case–control	979 cases and 14,666 controls	PM_10_	Autism spectrum disorder (ASD)	PM10 exposure during the third trimester was associated with ASD (OR = 1.36, 95% CI: 1.13–1.63)	Race, maternal education, maternal age, smoking in pregnancy, marital status, census tract median household income, and urbanicity
Raz et al. (84)	USA	Nested case–control	245 cases and 1,522 randomly selected controls	PM_10–2.5_ and PM_2.5_ concentration before, during, and after pregnancy estimated by validated spatiotemporal models	Autism spectrum disorder (ASD) assessed by parental report (validated)	PM_2.5_ exposure during pregnancy was associated with increased odds of ASD; with an adjusted odds ratio (OR) for ASD per interquartile range (IQR) higher PM_2.5_ (4.42 μg/m^3^) of 1.57 (95% CI: 1.22–2.03). The association between ASD and PM_2.5_ was stronger for exposure during the third trimester	Child’s birth year, birth month, and sex, maternal age at birth, paternal age at birth, and median census tract income in the birth year
Sunyer et al. (59)	Catalonia, Spain	Cohort	2,715 children aged 7–10 years	Elemental carbon (EC), NO_2_, and ultrafine particle number (UFP) measured by air monitors set at schools	Cognitive development assessed with the n-back and the attentional network tests	Children attending schools with higher levels of EC, NO_2_, and UFP both indoors and outdoors experienced substantially smaller growth in all the cognitive measurements	Age, sex, maternal education, socioeconomic status, and air pollution exposure at home
Talbott et al. (85)	Pennsylvania, USA	Case–control	217 cases and 226 controls	PM_2.5_ predicted by LUR during pre-pregnancy, trimesters one through three, pregnancy, years one and two of life and cumulative (starting from pre-pregnancy)	Causes of autism spectrum disorder (ASD) assessed by Social Communication Questionnaire (SCQ) or medical documentation.	An IQR increased in average exposure to PM_2.5_ (μg/m^3^) during year 2 postnatal (aOR: 1.45 (1.01–2.08)), and during pre-pregnancy through year 2 (aOR: 1.51 (1.01–2.26)) were associated with ASD.	Mother’s age, education, race, and smoking.
Talbott et al. (86)	Pennsylvania, USA	Case–control	217 ASD cases and 5231 controls	30 neurotoxicants assessed by US EPA National Air Toxics Assessment	Causes of autism spectrum disorder (ASD) assessed by Social Communication Questionnaire (SCQ) or medical documentation	Comparing fourth to first quartile exposures for all births, the adjusted OR for styrene was 1.61 (95% CI = 1.08–2.40, *p* = 0.018). Chromium also exhibited an elevated OR of 1.60 (95% CI = 1.08–2.38, *p* = 0.020)	Mother’s age, race, education, smoking, child’s birth year, and child’s sex
Calderón-Garcidueñas et al. (49)	Mexico City Metropolitan Area (MCMA) and small cites in Mexico (Zacatlán and Huachinango, Puebla; Zitácuaro, Michoacán; Puerto Escondido, Oaxaca)	Case-control	Children cohort from MC and control location consisting of small cities in Mexico	Exposure to high concentrations of air pollutants, i.e., PM_2.5_ and ozone	Impact on neurovascular unit dysfunction and risk of Alzheimer’s disease	Major findings in MC residents included leaking capillaries and small arterioles with extravascular lipids and erythrocytes, lipofuscin in pericytes, smooth muscle and EC, thickening of cerebrovascular basement membranes with small deposits of amyloid, patchy absence of the perivascular glial sheet, enlarged Virchow–Robin spaces and nanosize particles (20–48 nm) in endothelial cells, basement membranes, axons and dendrites. The integrity of the neurovascular unit, an interactive network of vascular, glial and neuronal cells is compromised in MC young residents	
Chiu et al. (62)	Boston, MA, USA	Cohort	267 singleton full-term urban children from ACCESS project	Exposure to prenatal particulate air pollution	Neurodevelopment in urban children	The study suggests the associations between higher PM_2.5_ levels at 31– 38 weeks with lower IQ, at 20–26 weeks gestation with increased OEs, at 32–36 weeks with slower HRT, and at 22–40 weeks with increased HRT-SE among boys, while significant associations were found in memory domains in girls (higher PM_2.5_ exposure at 18–26 weeks with reduced VIM, at 12–20 weeks with reduced GM). Increased PM_2.5_ exposure in specific prenatal windows may be associated with poorer function across memory and attention domains with variable associations based on sex	Maternal age, race, and educational status, child’s sex, date of birth, parity, gestational age at birth, and birth weight, smoking status, breast feeding duration, and children’s blood lead levels
Forns et al. (52)	Barcelona (Catalonia, Spain)	Cross-sectional	2897 children 7–11 years of age from 39 schools in Barcelona, during 2012–13	Exposure to traffic-related air pollution and noise at school	Behavioral problems in school-age children	Exposure to TRAPs at school was associated with increased behavioral problems in schoolchildren. Noise exposure at school was associated with more ADHD symptoms. Interquartile range increases in indoor and outdoor EC, BC, and NO2 concentrations were positively associated with SDQ total difficulties scores (suggesting more frequent behavioral problems) in adjusted multivariate models, whereas noise was significantly associated with ADHD-DSM-IV scores	Child age and sex, maternal and parental education, maternal occupation, socioeconomic status, sibling at birth, smoking and alcohol consumption during pregnancy, duration of breastfeeding, BC concentrations at home, traffic noise annoyance at home, home tobacco use, the Urban Vulnerability Index at home address, and the area level
Grineski et al. (54)	El Paso, Texas, USA	Cross-sectional	Caretakers of 1888 fourth and fifth grade children in the EPISD	School-based exposure to hazardous air pollutants	Impact of individual children’s academic performance (Grade point average)	An interquartile range increase in school-level HAP exposure was associated with an adjusted 0.11–0.40-point decrease in individual students’ grade point averages (GPAs). Non-road mobile and total respiratory risk had the largest effects on children’s GPA of all HAP variables studied	Age, sex, race/ethnicity, English proficiency, mother’s education, economic deprivation, percent of students economically disadvantaged and student-teacher ratio
Guxens et al. (87)	Europe (Sweden, Netherlands, Italy and Spain)	Population-based cohort	8079 children	Exposure to air pollution during pregnancy	Childhood autistic traits in the general population	Prenatal air pollution exposure was not associated with autistic traits within the borderline/clinical range (odds ratio = 0.94; 95% CI: 0.81–1.10 per each 10-μg/m3 increase in NO2 pregnancy levels). Similar results were observed in the different cohorts, for the other pollutants, and in assessments of children with autistic traits within the clinical range or children with autistic traits as a quantitative score	Age at delivery, education level, country of birth, prenatal smoking, parity, maternal height, pre-pregnancy weight, child sex and date of birth, and child’s age at autistic trait assessment
Porta et al. (70)	Rome	Prospective Cohort	719 newborns delivered in two large obstetric hospitals	Early life exposure to (traffic-related) air pollution	Cognitive impairment in children	A 10 μg/m^3^ higher NO_2_ exposure during pregnancy was associated with 1.4 fewer points (95% CI: −2.6 to −0.20) of verbal IQ, and 1.4 fewer points (95% CI: −2.7 to −0.20) of verbal comprehension IQ. Similar associations were found for traffic intensity in a 100 m buffer around home	Gender, child age at cognitive test, maternal age at delivery, parental and maternal educational level, siblings, socioeconomic status, maternal smoking and alcohol consumption during pregnancy, BMI, area-based socioeconomic index, and birth weight
Yorifuji et al. (72)	Japan	Longitudinal	Singleton births between January 10 and 17 or July 10 and 17, 2001 throughout the country (*N* = 33911)	Prenatal exposure to (municipality-level) traffic-related air pollution	Child behavioral development milestone delays	Air pollution exposure during gestation was positively associated with the risk of some developmental milestone delays at both ages (2.5 & 5.5 years.). Air pollution associated with verbal and fine motor development at age 2.5 years; and with behaviors related to inhibition and impulsivity at 5.5 years. ORs following one interquartile-range increase in nitrogen dioxide and suspended particulate matter were 1.24 (95% CI: 1.07–1.43) for inability to compose a two-phrase sentence at ages 2.5 and 1.10 (1.05, 1.16) for inability to express emotions at age 5.5 years	Sex and birth of children, mean maternal age at delivery, parity, maternal smoking status and educational attainment, mean paternal income, residential area, per capital income tax, and population density

### Air Pollution and Neurotoxicity in Adults

#### Effects on Brain Biology and Histology

Histological and biological changes in the CNS in human after exposure to ambient air pollution have rarely been studied, perhaps due to the cost associated with complex study procedures. Two studies examined the association between air pollution and human brain inflammation (24, 26), and two controlled human exposure studies investigated the effect of exposure to diesel exhaust on the CNS (25, 27). The changes of expression of cyclooxygenase-2 (COX2) and accumulation of the 42-amino acid form of β-amyloid (Aβ42), which are two common inflammatory markers, were examined in human brain samples from lifelong residents of large cities with severe air pollution (exposed) and control subjects from small cities with low air pollution levels (unexposed) in Mexico. The studies reported significantly higher COX2 expression and greater neuronal and astrocytic accumulation of Aβ42 among residents of cities with severe air pollution compared to residents in low air pollution cities (24, 26). The authors contend that high COX2 expression and Aβ42 accumulation are characteristics of neurological disorders, such as Alzheimer’s disease, suggesting that air pollution may increase the risk of neurological disorders. Despite significant findings and the important contribution to the field as one of the few studies investigating the link between air pollution and brain histology, the results of these studies need to be interpreted with caution because of some limitations. Specifically, these studies have very small, convenient sample sizes of 19 and 59, respectively. The low sample sizes threaten the studies’ validity and prevent them from making generalization. People who were selected to be in the studies may not be representative of the whole population in high and low air pollution exposure areas under study. Second and most importantly, the studies do not have a clearly defined independent variable to be tested. They examined two groups of people from high and low pollution areas; however, high and low pollution were not objectively defined, making it highly subjected to exposure misclassification since personal exposure could vary from person to person despite living in the same area. In other words, a person living in a low exposed city may have higher personal exposure level. In addition, the analyses were not adjusted for important factors besides pollution that could have explained the differences in brain histology found in the studies.

In an attempt to minimize threats to validity, such as confounding and misclassification between comparison groups, Cruts et al. (25) conducted a double-blind randomized crossover study to examine the relationship between exposure to dilute diesel exhaust and changes in brain activity measured by quantitative electroencephalography. Diesel exhaust was delivered for 30 min by an engine which produced 300 μg/m^3^ of suspended particles, 1.6 ppm nitrogen oxide, 4.5 ppm nitric oxide, 7.5 ppm carbon monoxide, and 7.5 ppm total hydrocarbon. The authors found a significant increase in median power frequency in the frontal cortex after 30 min of exposure to diesel exhaust. This study suggests that diesel exposure can lead to a general cortical stress response (25). However, Cliff et al. used the same study design to examine the acute effects of diesel exhaust exposure on biomarkers of systemic and CNS inflammation. No significant effects were observed among 27 healthy adults (27). In these two studies, confounding is not likely a major issue because the comparison groups are the same subjects. However, again, it has limited generalizability due to a small sample size.

Overall, despite there are numerous studies showing exposure to air pollution can increase neuroinflammation in animal studies (88–91), evidence among human is still relatively limited and inconsistent. The need to examine brain tissues and activities prevents human studies from being abundant. Additionally, the few existing studies suffer from small sample size issues because it is not feasible to conduct brain histological studies at a population level.

#### Effects on Neuropsychological Function

##### Evidence from the Cross-Sectional Studies

Studies for neuropsychological effects have been more popular and vary in study design and methodology. Several cross-sectional studies have examined the associations between ambient or TRAP and neurobehavioral functions or neurological symptoms in adults. They suggest higher exposure to air pollution is associated with lower performance in neuropsychological tests. For example, Inserra et al. (33) examined the long-term health effects of ambient hydrogen sulfide (H_2_S) exposure – determined by universal Kriging of known observations from air monitors – on neurobehavioral performance among 335 residents in more clearly defined exposed (>90 bbp) and unexposed (<50 bbp) neighborhoods in Dakota City, Nebraska. This study reported that after adjusting for age, gender, and testing language in a multivariate logistic regression model, poorer performance on a test of memory was marginally associated with high H_2_S-exposed residence [odds ratio (OR) = 0.6, 95% confidence interval (CI) = 0.4–1.0], while other neurobehavioral functions (e.g., motor response, mood/affect) were not associated with exposure (33). In addition to having only marginally significant lower odds of having better memory test performance in the exposed group, the study might have suffered non-response bias. For example, not only the generalizability of the study was threatened by the fact that participation rate was only less than 75%, non-respondent rate was higher in the unexposed group. This could have biased the results either toward or away from the null depending on the characteristics of non-respondents. Most importantly, this study is also subjected to exposure misclassification bias because not only it estimates exposure indirectly using statistical model but it also ignores personal activity patterns, which could have influenced personal exposures. As results, people who live in low exposed neighborhood may in fact have high exposure levels, and vice versa.

Chen and Schwartz (31) also examined neurobehavioral effects of long-term exposure to ambient PM and ozone in adults using the Third National Health and Nutrition Examination Survey (NHANES III) conducted in 1988–1994. Exposure was estimated using distance-weighted averages from all monitors in the residing and adjoining counties. After adjustment for important confounding variables, such as sociodemographic factors, lifestyle factors, medical risk factors, place of residence, and indoor pollution a in multiple linear regression analysis, each 10-ppm increase in annual ozone exposure was associated with increased symbol-digit substitution test score (SDST) and serial-digit learning test (SDLT) by 0.12 (95% CI: 0.01–0.23) and 0.57 (95% CI: 0.08–1.06), respectively. Since highly exposed individuals had higher scores, indicating slower reaction time, these findings suggest adverse neurobehavioral effects of ambient air pollutants in adults (31). No significant findings were observed for PM_10_. In this study, the exposure assessment only uses one-time residential information based on environmental monitoring data; therefore, it might not have captured an accurate exposure measurement for individuals, leading to possibly inaccurate estimate of risk. In addition, the cross-sectional nature of the study prevents a causal interpretation due to the lack of temporality, although it is not likely that people with lower test scores chose to live in area with higher ozone concentration.

Ailshire and Crimmins (29) examined the cross-sectional association between residential concentrations of PM with aerodynamic diameter of 2.5 μm or less (PM_2.5_) and cognitive function in older adults using multilevel linear regression models. Older adults living in areas with higher PM_2.5_ concentrations had worse cognitive function (β = −0.26, 95% CI: −0.47 to −0.05) even after adjustment for community- and individual-level social and economic characteristics. The strengths of the study are including a large, nationally representative sample of US older adults and considering individual and neighborhood confounders (29). Ailshire and Clarke (28) also conducted a similar cross-sectional association on neighborhood-level exposure to PM_2.5_ and cognitive function in a diverse, national sample of older U.S. adults (*n* = 780). The study found that those living in areas with greater exposure to PM_2.5_ had an error rate 1.5 times greater than those exposed to lower PM_2.5_ concentrations (IRR = 1.53, 95% CI: 1.02–2.30) for 10-μg/m^3^ increments (28). For these two studies, the limitations include lacking of some components of cognitive assessments; neighborhood based measure of pollution may not fully apprehend individual-level exposure; being unable to determine long-term exposure to air pollution on cognitive function; missing other potentially important confounders, such as diet and lifestyle factors.

Gatto et al. (32) also examined cross-sectional associations between various ambient air pollutants (O_3_, PM_2.5_, and NO_2_) and cognitive function among 1,496 adults (mean age 60.5 years) living in the Los Angeles Basin using regression model and found that 10-μg/m^3^ increases in PM_2.5_ was significantly associated with lower verbal learning (β = −0.32, 95% CI: −0.63 to 0.00; *p* = 0.05) and significant association was observed with global cognition, after adjusting for age, gender, race, education, income, study, and mood. NO_2_ exposure >20 ppb and O_3_ ≤34 ppb was related with lower logical (β = −0.62, 95% CI: −1.35 to 0.11, compared to ≤10 ppb) and executive function (β = −0.66, 95% CI: −1.35 to 0.03). The strength of this study provides addition evidence of the effects of gaseous air pollutants on cognitive function. However, measurement error in exposure assignments and a feature of cross-sectional study design limit this study (32).

Recently, Tzivian et al. (38) conducted another cross-sectional study of adverse effects of long-term air pollution on cognitive functions [mild cognitive impairment (MCI), amnestic MCI (aMCI) and non-amnestic MCI (naMCI)] among adults aged 45–75 years in highly urban German Ruhr area. Cognitive functions were assessed at 5 year follow-up examination of the population-based Heinz Nixdorf Recall study and exposure assessment used the LUR model. The study found that long-term exposure to air pollution were associated with MCI, particularly aMCI (PM_2.5_: OR = 1.22, 95% CI: 1.08–1.38). The main strength of this study is its capability to distinguish the effects of TRAP from traffic noise. In addition, large study sample, standard assessment method for outcome measurement, and availability of other confounders, such as lifestyle factors and medical history, are other strengths. However, exposure misclassification and cross-sectional study feature limit the value of this study in the causal inference (38).

##### Evidence from the Cohort Studies

To address the weakness of cross-sectional studies, several cohort studies have been conducted to examine the effects of air pollution exposure in the period preceding cognitive testing. For example, Ranft et al. examined the health effects of long-term exposure to TRAP on cognitive impairment in elderly women aged 68–79 years in Germany. The study found that living near busy streets within 50 m was associated with mild cognitive impairment measured by the neuropsychological test battery CERAD-Plus test, stroop test, and sniffing test after adjusting for important covariates, such as patient demographic factors, lifestyle, and medical history, in linear regression models (36). The main limitation that one needs to notice is the fact that although only people who lived in the same area for 20 years or more are included, activity patterns were not adjusted for. Residential exposure might not be accurate exposure measurement since people could spend most of their time elsewhere that has different exposure levels. A similar study in the U.S. also found similar results with significantly decreasing cognitive function scores with closer proximity to major road ways after adjustment for important covariates (Table 1) (39).

In addition, the effects of exposure to PM_2.5–10_ on cognitive decline were recently evaluated among 19,409 older women aged 70–81 years, who were part of the Nurses’ Health Study cohort study (40). Recent and 7–14 years exposures to PM preceding baseline cognitive assessment were estimated using spatiotemporal smoothing models with the US EPA air quality monitoring system. This study found that higher long-term exposure to both PM_2.5–10_ and PM_2.5_ are associated with faster two-year cognitive declines after adjusting for important covariates in a generalized estimating equation regression model (Table 1). Despite significant evidence, similar to the previous study, the results of this study might be biased by exposure misclassification since estimation was indirect using spatial modeling, which may not adequately adjust for personal activity patterns.

Loop et al. examines the 1-year mean PM_2.5_ concentration preceding the cognitive assessment that was measured over the telephone using the Six-Item Screener (SIS) in a biracial, bigender national cohort (*n* = 20,150) of at least 45 years of age. The study found that only 8% were classified as the cognitively impaired in their most recent follow-up. A 10-μg/m^3^ increase in PM_2.5_ concentration was not reliably associated with an increased odd of incident impairment (1.26, 95% CI: 0.97–1.64), but was slightly associated with incident impairment in urban areas (1.40, 95% CI: 1.06–1.85). The strengths of the study include a nationwide and demographically diverse US cohort and satellite measurements for air pollution exposure assessment. Its major limitations could be a potential of systemic misclassification of outcome and exposure measurements and irrelevant windows of exposure (34).

Tonne et al. investigated the association between exposures to particulate air pollution (characterized by size and source) and a cognitive battery composed of tests of reasoning, memory, phonemic and semantic fluency, among the participants from the Whitehall II longitudinal cohort study (*n* = 2867). All particle metrics were associated with lower scores in reasoning and memory measured in the 2007–2009 wave but not with lower verbal score. Higher PM_2.5_ of 1.1 μg/m^3^ (lag 4) was associated with a 0.03 (95% CI: −0.06 to 0.002) 5-year decline in standardized memory score and a 0.04 (−0.07 to −0.01) decline when restricted to participants remaining in London between study waves. This study provides support for an association between particulate air pollution and some measures of cognitive function, as well as decline over time in cognition; however, it does not support the hypothesis that traffic-related particles are more strongly associated with cognitive function than particles from all sources. This study included relatively large population cohort residing in largest city of Europe and explored various windows of exposure preceding cognitive testing (37).

##### Evidence on Chemical Constituents of PM

Studies of the effects of chemical constituents of PM on cognitive function in adults are limited. Only two studies have been found. One study reported that black carbon (BC) exposure was associated with declined cognitive functions among older men in another recent study (35). In their study, Power et al. found that for each doubling in daily average BC concentration measured by LUR models based on local monitor sites, the odds of having mini-mental status examination (MMSE) scores under 25, which indicates cognitive impairment, increases by 30% after adjustment for important covariates (Table 1). Each doubling in BC concentration was also associated with a 0.054 SD lower cognitive test scores (95% CI, −0.103 to −0.006). This study, however, also used indirect exposure estimation through mathematical models and did not adjust for personal activity patterns among adult elderly subjects.

The other cross-sectional study examined the effects of airborne manganese (Mn) from industrial sources on cognitive function of adults residing in two Ohio towns. Significant relationships (*p* < 0.05) between Mn exposure and performance on working and visuospatial memory (e.g., Rey-O Immediate *b* = 0.19, Rey-O Delayed *b* = 0.16) and verbal skills (e.g., Similarities *b* = 0.19) were found. The strength of the study includes use of stringent selection criteria as participants with prior or present occupational exposures to chemicals or neurotoxic agents at work were excluded; tests were administered by clinically trained administers and use of advanced modeling. The limitations of the study are absence of personal sampling of each participant’s exposure to Mn; the estimation of Mn inhalation exposure varies depending on the Mn release characteristics in both towns and uncertainties in air dispersion modeling; and bias may occur as participants were not randomly selected in those towns (30).

Overall, studies investigating the effects of various air pollution measures on neuropsychological functions are more common; however, their methodologies vary significantly, leading to difficulty in direct comparison between studies. First, it is important to recognize that individual exposure may be very different from air pollution levels recorded by air monitors. In fact, none of the studies we reviewed have adjusted for personal activities patterns. Secondly, studies have focused on different air pollutants with different methods of assessment, most of which are indirect estimates using models or nearby air monitors. Therefore, although there is evidence on general effects of air pollution, this evidence is not for yet sufficient any one particular pollutant. In addition, the studies also used various neuropsychological tests for the same construct, which make comparisons difficult. Moreover, lifetime air pollution exposure assessment preceding cognitive testing is challenge. Research on more appropriate methods is needed.

Despite weaknesses and some mixed findings, there is balance of evidence suggesting that air pollution has adverse effects of neuropsychological functions as measured by test scores in adults. More research is still warranted given the heterogeneity of current research.

#### Effects on Neurodegenerative Disorders

Several studies have been conducted to examine the effects of air pollution or TRAP on neurodegenerative disorders, such as Parkinson’s disease, dementia, Alzheimer’s disease, and others. Finkelstein and Jerrett, in a retrospective cohort study, investigated the association between Parkinson’s disease and ambient air Mn in a cohort of 110,000 subjects in the cities of Toronto and Hamilton, Canada. Ambient Mn exposure was estimated by the Mn fraction of total suspended particulate (TSP) using two directly interpolated surfaces of TSP and Mn-enrichment factor. In Hamilton, the OR of Parkinson’s disease was found to be 1.034 (95% CI: 1.00–1.07) per 10 ng/m^3^ increase in Mn in TSP after controlling for sex and birth year group in multiple logistic regression models (43). No significant differences were observed in Toronto. A closer analysis of age suggested that exposure to Mn advances the age of diagnosis of Parkinson’s disease, indicating a significant negative effect. Although the magnitude of effect is relatively small, it might suggest clinical significance since Parkinson’s is a serious disease that urgently needs intervention reduction of risk. In addition, the lack of residential mobility adjustment and the fact that interpolation models for exposure assessment might not have adequately represented personal exposure could have affected the results. Despite these potential limitations and mixed results for two cities, this study has shown some evidence toward the negative effects of pollution on Parkinson’s disease.

Chang et al. evaluated the effects of air pollution on the risk of dementia using the data obtained from the National Health Insurance Research Database (NHIRD) of Taiwan, including those diagnosed with dementia between 2000 and 2010. Yearly average concentrations of pollutants were calculated from the baseline to the date of dementia occurrence, withdrawal of patients, or the end of the study. This study indicated that exposures to NO_2_ and CO were associated with an increased risk of dementia in the Taiwanese population. The limitations of this study include potential biases due to retrospective nature, unknown confounders, and unavailable relevant clinical variables since NHIRD data are anonymous. Large sample size, long follow-up period, and the reliability and accuracy of diagnosis and codes of dementia from the NHIRD are considered as the strengths of this study (42). Oudin et al. also conducted a prospective cohort study to examine the effects of air pollution on dementia in Northern Sweden. They also found that participants with highest TRAP exposure was associated with dementia diagnosis (hazard ratio (HR): 1.43, 95% CI: 0.998–2.05) compared with those with lowest exposure. The study used the high quality data from the Betula study. However, this study did not consider the effects of traffic noise, which is highly correlated with TRAP (47).

Heydarpour et al. examined the spatial distribution of prevalent multiple sclerosis (MS) cases and their association with the spatial patterns of air pollution assessed with the previously developed LUR models. This study suggests that there was a statistically significant difference in exposure to PM_10_, SO_2_, NO_2_, and NOx (*p* < 0.001) in MS cases compared with controls. One of the main limitations of the study design is that no confounder was considered in the analysis (44). Malek et al. conducted a case–control study (51 cases, 51 controls) to examine the associations between suspected HAPs exposure and ALS in 6 counties near Philadelphia, from 2008 to 2011. Exposure to HAPs was assessed using residential census tract level data from the U.S. EPA National-Scale Air Toxics Assessment (NATA) data. This study showed that residential exposure to ambient air aromatic solvents was associated with increasing risk of ALS in 2002 (OR: 5.03, 95% CI: 1.29–19.53) and 1999 (OR: 4.27, 95% CI: 1.09–16.79). The study had a small sample size and exposure misclassification due to ecological measurements of exposure to HAPs and residential change (46). Angelici et al. examined the association of exposure to airborne PM on the occurrence of MS-related hospitalizations in Italy during 2002–2009. A total of 8287 MS-related hospitalization was obtained through discharge records and exposure to air pollutants was measured via air monitoring stations. Using Poisson regression, it was found that higher risk of MS hospital admission was associated with exposure to PM_10_ and the highest effect was observed from 0 to 7 days. A major limitation of the study is the feature of ecological study design (41).

Jung et al. conducted a cohort study of 95,690 individuals’ age ≥65 years old during 2001–2010 to determine the association between newly diagnosed Alzheimer disease and long-term exposure to ozone (O_3_) as well as PM_2.5_ in Taiwan. The findings suggest that long-term exposure to O_3_ and PM_2.5_ above the current US EPA standards were associated with increased the risk of Alzheimer disease. The strengths of the study include the use of population-based database; of the use of newly diagnosed cases; and the first study conducted in Asian population. However, the major limitations include exposure errors of air pollution assessment and inadequate controlling of confounding (45).

Overall, the studies of air pollution and neurodegenerative disorders are limited. Existing studies have the limitations of poor air pollution exposure assessment and inadequate control of confounders. Relevant windows of exposure, more studies are needed to determine the association between air pollution and neurologically related disorders, such as Parkinson’s, Huntington, and Alzheimer’s diseases with appropriate methods.

#### Effects on Other Non-Specific Neurological Symptoms

A cross-sectional study examined the relationship between ambient ozone and building-related symptoms based on the U.S. EPA’s Building Assessment Survey and Evaluation Study data. Ambient ozone level was estimated using the nearest US EPA local air monitors. This study found that neurological symptoms of fatigue and trouble concentrating were significantly associated with ambient ozone (OR = 1.03, 95% CI: 1.01–1.05 per 10 μg/m^3^ increase in late workday ambient ozone) after adjusting for personal, workplace, and environmental variables, such as age, gender, smoking status, allergy, thermal exposure, humidity, and season in logistic regression models. Headache was also associated with ambient ozone (OR = 1.03, 95% CI: 1.00–1.05 per 10 μg/m^3^ increase in late workday ambient ozone) after controlling for the same covariates previously mentioned (48). Although the ORs for association between ozone and neurological symptoms and headache are statistically significant, it is worth noticing that they are relatively small in magnitude. This study is limited by the cross-sectional design, which does not provide information on whether exposure precedes outcome or vice versa. In addition, the fact that ozone exposure was estimated using air monitors at varying distance away from buildings could limits the accuracy of exposure measurement. Furthermore, using study space averages of environmental exposure to assign to individual exposure could also hinder accuracy because it does not take into account personal activities.

Epidemiological evidence on adverse health effects of ambient air pollution on the nervous system in adults is generally limited. However, given what is available, evidence from the reviewed studies – regardless of study design – generally suggest that ambient air pollution has potentially adverse effects on neurobehavioral functions. However, it warrants further investigation with more advanced study designs, such as more cohort studies that allow for examination of temporal sequence, and better exposure assessment for more accurate estimation of risk. Since most studies reviewed above [except Ranft et al. (36), Chen and Schwartz (31), and Weuve et al. (40), which adjusted for education and income] were not able to adjust for community level factors (e.g., median income, housing status, etc.) and the gene–environment interaction – two factors which are known to be associated with both exposure and outcome – it is very important for future studies to also adjust for these factors to get more accurate estimation of effects (92, 93). Studies that show statistically significant effects of air pollution generally show weak magnitude of association. This could be because of the inability to control for these factors, which could be strong confounders since they may have stronger effect on neurobehavioral outcomes compared to air pollution. Furthermore, as previously mentioned, methodologies, including pollutant of interest, exposure assessment, and outcome assessment, vary between studies. This makes it difficult to directly compare results.

### Air Pollution and Neurotoxicity in Children

#### Effects on Brain Biology and Histology

Similar to adults, studies that examine the effects of air pollution on brain histology and biology in children are rare. This is perhaps due to the complex nature of study procedures and the difficulty in obtaining participants, especially for population level studies. Three studies were conducted by the same research group to evaluate the health effects of air pollution on brain abnormalities measured by magnetic resonance imaging (MRI), immuno-histochemistry and electron microscopy among children in Mexico. Residency in a highly exposed area (Mexico city) was significantly associated with a higher proportion of prefrontal white matter hyperintense lesions compared to low exposed area (Polotitlán) in a hierarchical regression model adjusted for age and gender (23, 49). The pathological examination of olfactory bulbs (OB) pathology examined by immuno-histochemistry and electron microscopy also showed that the exposed groups exhibited immunoreactivity to Aβ42 and α-synuclein and OB endothelial hyperplasia, and neuronal accumulation of particles, suggesting more neuroinflammation (22).

Despite positive findings, these three studies are limited from the cross-sectional design, small sample size, and the fact that important confounders (e.g., other health conditions for the first study, the latter study did not adjust for any confounder) were not adjusted for, making inference particularly weak. Most importantly, the independent variable in this study is not clearly indicated, suggesting that one cannot attribute the reported effects to pollution. Specifically, the authors have classified people in Mexico city as highly exposed while those in Polotitlán as control. This might have been subjected to misclassification of exposure because the level of exposure within city can vary depending on activity patterns and local variation.

Overall, evidence on the effects of pollution on brain biology and histology is still limited. Existing studies show evidence of adverse effects; however, are limited from methodological weaknesses, such as small sample size, lack of adjustment for potential confounders, poorly defined exposures, etc. More studies with more rigorous methods are still needed to yield clearer conclusion in this area.

#### Effects on Child Neurodevelopment

Studies for neuropsychological functions are more common. A total of 24 studies have been conducted to examine health effects of air pollution or TRAP during pregnancy and/or early childhood on neurodevelopment in children.

##### Prenatal Exposure to Air Pollution Alone

Fetuses are more susceptible to the harmful effects of a variety of environmental contaminants. Prenatal exposure to air pollutants may produce a variety of neurodevelopmental problems as a result of irreversible nervous system damage. Due to this concern, several epidemiological studies have been developed to examine prenatal exposure to air pollution on early child neurodevelopment.

Perera et al. conducted a prospective cohort study to examine the effects of prenatal exposure to air polycyclic aromatic hydrocarbons (PAHs) on neurobehavioral disorders among 183 subjects. Personal monitoring during the third trimester of pregnancy was used to collect the air samples, which were analyzed for prenatal exposure to airborne PAHs, and the Bayley Scales of Infant Development-Revised version was used to assess mental and psychomotor development in the first 3 years of life. The study found that children in the upper quartile of exposure to PAHs during the third trimester scored 5.69 points lower using the mental development index than those in the lowest quartile of exposure to PAHs. In addition, the risk of cognitive developmental delay for high PAHs exposure group was almost three times compared to children in the low exposure group (69). The most important limitation this study has, as recognized by the authors, is the fact that while postnatal exposures and exposure during other times of pregnancy can potentially affect the outcome, they were not available for adequate adjustment. Similarly, Tang et al. evaluated the associations between prenatal exposure to ambient PAHs, lead, and mercury from combustion of coal and fossil fuels, and cognitive functions measured by the Gesell Developmental Schedules at 2 years of age among children in Tongliang and Chongqing, China. PAH–DNA adducts and lead and mercury in blood cord were measured as individual exposure to these chemicals. After adjusting for important confounders, increased PAH–DNA adducts was associated with decreased motor area developmental quotients (DQ) and average DQ (Table 2). The OR of developmental delay in motor area was 1.91 (95% CI: 1.22–2.97) per 0.1 unit increase in PAH–DNA adduct (71). Similar to the previous study, the authors recognized that the lack of postnatal exposure could be problematic because these exposures may have effect on subsequent development. While the use of biomarkers for measurement of exposure is less subject to misclassification, studies like these could still be limited by the fact that it assumes peripheral biomarkers are accurate representations of what is going in the CNS. Nevertheless, with more rigorous methodology including prospective cohort design, more accurate exposure assessment via biomonitoring, and adjustment of potential confounders, the association between ambient air pollution and adverse neurological effects remains consistent. Lovasi et al. also examined the effects of prenatal exposure to PAH and neighborhood social context on cognitive test scores at 5 years of age measured by the Wechsler Preschool and Primary Scale of Intelligence-Revised (WPPSI-R) in 1998–2006. Prenatal PAH exposure during the third trimester of pregnancy was measured via personal monitoring. The study found that the prenatal PAH exposure were significantly associated with 3.5-point lower WPPSI-R total and 3.9-point lower verbal scores. Some strengths of this study include prospective design, the presence of data from prenatal personal PAH, and neighborhood definition using 1-km network buffers, etc. However, high-risk women before the third trimester and children who took Spanish version of the test scores were excluded from the study, thus, may result in selection bias (68). Another recent longitudinal study was also conduct to investigate the association between prenatal PAH exposures and cognitive dysfunction in 170 children in Krakow, Poland. The exposure was also assessed using the cord blood PAH–DNA adducts. Consistent findings with Tang’s study were observed. This study is limited due to relatively smaller number of subjects in the study and the main strength is individual-level prenatal exposures to PAH, based on cord blood PAH–DNA adducts (66).

Guxens et al. examined the associations between prenatal exposure to residential air pollution assessed by LUR and infant mental development among 1889 children. The study also showed negative effects of exposure to ambient air pollution on neuropsychological outcomes measured by Bayley Scales of Infant Development. Specifically, the study showed that prenatal exposures to NO_2_ and benzene were inversely and significantly associated with infant mental development in the group of low intake of vegetable and fruit during pregnancy (64). In this study, loss to follow-up was more likely to be participants from lower socioeconomic class, which could lead to bias; however, in analyses, the authors included large sets of variables related to participation and results remained consistent across strata. Therefore, in addition to showing that prenatal exposure to residential air pollution negatively affects mental development, this study also shows potential effect modification of the level of antioxidant intake. In other words, antioxidant intake can alleviate the negative effects of air pollution on mental development. Furthermore, Guxens et al. examined prenatal exposure to outdoor air pollution and its effect on cognitive and psychomotor development in childhood (*n* = 9482) within six prospective cohort studies. The concentrations of air pollution were also calculated using LUR models, air monitoring campaigns and back-extrapolation procedure. Similarly, a negative association was observed between prenatal air pollution, especially NO_2_ in psychomotor development in children aged 1–6 years but no association was observed in cognitive development. The strength of this study provides evidence on long-term effects of prenatal exposure on neurodevelopment among the pooled samples of several large cohorts. The main limitations include the heterogeneity of outcome measurements from different cohort and exposure measurement errors (65).

In South Korea, a prospective birth cohort study was conducted to study the effects of prenatal exposure to air pollution (PM_10_ and NO_2_) on neurodevelopment in early childhood (first 24 months). The neurodevelopment was assessed using Korean Bayley Scale of Infant Development II (K-BSID-II) at ages of 6, 12, and 24 months and air pollutants exposure was measured using inverse distance weighting (IDW) model. Prenatal exposure to PM_10_ and NO_2_ was significantly associated with Mental Developmental Index and Psychomotor Developmental Index at 6 months of age, but no significance at 12 and 24 months of age. This study provided a longitudinal assessment of neurodevelopment at multiple ages while a relatively small sample size and air pollution exposure errors could limit the value of this study (67).

Cowell et al. investigated the effects of prenatal traffic-related BC exposure on children’s memory and learning. Validated spatiotemporal LUR models were used to calculate BC exposure. The Wide Range Assessment of Memory and Learning-Second Edition (WRAML2) was used to assess memory functions in children at 6 years of age. It was found that boys born to mother with the highest prenatal stress and higher BC showed lower memory score. The major strengths of the study are its prospective study design and inclusion of mother–child pairs from the understudied group with exposure to both air pollutants and stress. Similarly, this study has the limitations of exposure errors and residual confounding (63).

Chiu et al. examined sensitive windows of prenatal exposure to PM_2.5_ on neurodevelopment of children. Individual prenatal exposure to PM_2.5_ was measured using hybrid satellite-based spatio-temporal resolved prediction model and children aged 6.5 ± 0.98 years were tested for neurodevelopment measurement. This study found that increased PM_2.5_ exposure in specific prenatal windows was associated with poorer functions across memory and attention domains. The strengths of this study include the use of satellite data for air pollution exposure assessment and distributed lag models to identify sensitive windows for effects on neurodevelopment (62).

A recent Japanese study was also conducted to examine the association between prenatal exposure to outdoor air pollution and delay in behavioral development in nationally representative (singleton births) children. Positive associations between air pollution and neurodevelopmental outcomes were observed. Several limitations include the use of untested survey questions and methods of outcome measurements, and the possibility of misclassification errors (72).

Porta et al. conducted a prospective cohort study of TRAP and cognitive function among 719 newborns enrolled in GASPII project in Rome, where cognitive development of 474 children at age 7 were scaled using Wechsler intelligence Scale for Children-III and LUR model was used to assess exposure to NO_2_, PM_coarse_, PM_2.5_ and PM_2.5_ absorbance at birth. This study showed that prenatal exposure to NO_2_ per 10 μg/m^3^ increase was inversely associated with the verbal IQ (−1.4, 95% CI: −2.6, −0.20) and verbal comprehension index (−1.4, 95% CI: −2.7, −0.20). In addition, it was also found that traffic intensity of all roads in a 100-mt buffer and total traffic intensity of major roads in a 100-mt buffer were inversely associated with full-scale IQ, Verbal IQ and Verbal comprehension index. The study uses standardized measures to calculate the exposure for each pollutant. The study was limited due to selection bias at the enrollment period as the study participants were reprehensive of more educated group and loss of follow-up was caused by socioeconomic status (SES) status (70).

Overall, existing evidence suggests that prenatal exposure to air pollutants may have impacts on child neurodevelopment regardless different study designs, study populations, air pollution exposure assessments, and outcome measurements. Although most of studies rely on the data from air monitors or statistical methods for air pollution exposure assessments, a few other studies have been conducted to investigate the neurological effects of exposure to ambient air pollution measured by more accurate and objective exposure assessment, such as biomarkers in children. More research on identifying sensitive windows and susceptible groups, application of objective exposure measurements, such as personal monitor, and intervention is required.

##### Prenatal and/or Childhood Air Pollution Exposure

Several studies have been conducted to assess the impacts of air pollution assessed at ecological levels, such as school or city levels, on cognitive functions in children. Calderón-Garcidueñas et al. investigated the effects of living in highly exposed city on cognitive functions among children measured by the Wechsler Intelligence Scale for Children-Revised. Residency in a highly exposed area (Mexico City) was significantly associated with cognitive deficit (23, 50). Furthermore, the same authors, in a subsequent study with the same exposure definition, examined the health effects of air pollution on olfactory dysfunction measured by the University of Pennsylvania Smell Identification Test (UPSIT). Results suggested that the highly exposed group had a significantly lower UPSIT score than the controls (22). As previously mentioned, these two studies are limited by sample size, poorly defined independent variable, and the lack of control for potential confounders. With larger sample size, a study conducted in China examined the health effects of TRAP on neurobehavioral functions among 282 third-grade children. A school in an area with severe TRAP was compared to another school from an area of low traffic density based on ambient air quality monitoring data. The level of traffic air pollution for the schools was determined by annual concentrations of nitrogen dioxide and PM_10_ measured by air monitors and on-site samplers. In multiple ordinal logistic regression models, after adjusting for other covariates, such as demographics, birth weight, delivery method, breast feeding, vision, familiarity with computer games, and household pollution, TRAP exposure was significantly associated with poorer performance on neurobehavioral tests (Table 2) (61). Van Kempen et al. conducted a cross-sectional study to investigate the association between air pollution and transportation noise on the cognitive performance from 553 children from 24 primary schools and in home setting, using multilevel modeling and found that NO_2_ exposure at school was statistically significantly with memory span length during DMST (*x*^2^ = 6.8, df = 1, *p* = 0.01). The combined interaction between air pollution and traffic noise at school was significantly associated with “block” condition of SAT; and at home, it was significantly associated with SRTT, Simple Reaction Time Test and ‘arrow’ condition of SAT (60). A prospective cohort study was conducted to examine the effects of TRAP exposure on cognitive development among 2715 children aged 7–10 years from 39 schools in Barcelona. TRAP at school was assessed. This study showed smaller growth in cognitive development in children at schools highly exposed to TRAP (7.4%, 95% CI: 5.6–8.8%) than low exposed schools (11.5%, 95% CI: 8.9–12.5%). Similarly, children at schools highly exposed to elemental carbon (EC), NO_2_, ultrafine particle number (UFP) had smaller improvements in all the cognitive parameters. (59). Grineski et al. also conducted a cross-sectional study to examine the impacts of school-level exposures to HAPs on academic performance at fourth and fifth grade children in the El Paso (Texas, USA). In multilevel models, HAP exposures at school were associated with lower individual-level grade point (54). Aside from previously mentioned weaknesses, such as inability to adjust for personal exposure and activity patterns, these studies are limited due to an ecologic exposure assessment.

In addition, a few cross-sectional studies have been conducted to provide useful information in this area. Kicinski et al. examined the association between traffic exposure and neurobehavioral performance in 606 adolescents in Belgium. Urinary levels of trans, trans-muconic acid, and the distance-weighted traffic density were used to estimated traffic exposures. The study indicated exposure to air pollutants was inversely associated with sustained attention (95% CI: −0.51 to −0.02). Similarly, no significant association was observed in traffic exposure and neurobehavioral domains. The study had some limitations, including a cross-sectional feature, the use of irrelevant urine samples as they weren’t collected at the time of examination, and the possibility of misclassification bias (56). In Spain, Forns et al. also conducted a cross-sectional study to examine the associations of exposure to TRAP (EC, BC and NO_2_) and noise at school on behavioral development of schoolchildren aged 7–11 years during 2012–2013. Forns et al. found similar results as described by Van Kempen et al. However, selection bias is one of concerns regarding this study (52). Overall, these studies limit their ability for causal inference due to the feature of cross-sectional study design.

Cohort study design has also been applied to study the impacts of air pollution on cognitive function in children. Suglia et al. examined the relationship between BC, measured by LUR models, and cognitive functions among 202 children in Boston, Massachusetts. Cognitive functions were measured by the Kaufman Brief Intelligence Test (KBIT) and the Wide Range Assessment of Memory and Learning (WRAML). After adjustment for age, gender, primary language spoken at home, mother’s education, birth weight, passive smoking, and blood lead levels, BC exposure was associated with a decreased scores in various cognitive measures (see Table 2) (58). Despite consistent association between exposure to pollution and risk of lower cognitive scores, results should be carefully interpreted. Specifically, this study assessed exposure at home residence while ignoring exposure at school/work at which participants might spend a considerable time, leading to inaccurate estimation of personal exposure and limited inference. Friere et al., in a cohort study investigated the effects of NO_2_ exposure on various neuropsychological constructs, such as general cognition, quantitative and working memory, and gross motor. Exposure assessment is more specific in this study and was measured by LUR model. Children exposed to higher NO_2_ (>24.75 mg/m^3^), compared to their counterparts, have a statistically significant decrease of 8.61 points in gross motor score, while there is no significant statistical differences for the other three constructs, which could likely be due to low sample size with too many covariates in analyses (Table 2) (53). Lin et al. recruited 533 mother-infant pairs from within 11 towns in Taiwan to study the effects of ambient air pollution on neurobehavioral development during pre and postnatal stages. This study found that increased SO_2_ exposure during pregnancy and post pregnancy (up to 1 year) was significantly associated with decrease in fine motor performance at 18 months Similar to other studies, residual confounding and exposure error are the main weaknesses of this study (57). Harris et al. studied associations between gestational and childhood exposures to TRAP and childhood cognition among a prospective cohort of 1,109 mother–child pairs in USA. The exposure to BC and PM_2.5_ were estimated using both residential proximity and validated spatiotemporal LUR models. The cognitive development was estimated using KBIT-2 at mean age of 8 years. Using linear regression model, lower non-verbal IQ (−7.5 points, 95% CI: −13.1 to −1.9), verbal IQ (−3.8 points, 95% CI: −8.2 to 0.6) and visual motor (−5.3 points; 95% CI: −11.0 to 0.4) scores were associated with residential proximity – 50 m away major roadway at birth than those living in ≥200 m away from roadway. PM_2.5_ exposure at third trimester had no association with poor cognition. One of the major limitations of this study is measure errors of exposure assessment (55). Cohort study design has the advantage of causal inference. However, similar to many studies discussed before, these studies are potentially subjected to exposure misclassification bias. More specifically, while children are not stationary, e.g., exposures were estimated using levels predicted at their home; therefore, this estimation may not reflect true exposure.

#### Effects on Neurodevelopmental Disorders

The potential impacts of air pollution on neurodevelopmental disorders, such as autism and attention deficit and hyperactivity disorder (ADHD), have arisen a great concern. Evidence on the association between air pollution and neurodevelopmental disorders has dramatically increased in recent years. To better test the causal relationship, several cohort and case–control studies have been conducted to examine the relationship between ambient air pollution and neurodevelopmental disorders in children.

##### Autism Spectrum Disorder

Windham et al. investigated the effects of HAPs on autistic spectrum disorder among children born in 1994 in San Francisco Bay area (76). Exposures to HAPs were determined by US EPA Gaussian air dispersion model. The study found that top quartiles of exposure to chlorinated solvents and heavy metals are associated with higher odds of having autistic spectrum disorder among children after adjusting for important covariates (Table 2). This study, however, is limited by the fact that exposure misclassification is possible due to indirect estimation by air dispersion model. More specifically, estimation of exposure at residential address using air monitoring sites cannot adequately control for personal activity patterns, leading to misclassification of exposure.

Kalkbrenner et al. also conducted a case–control study investigating the effect prenatal exposure to HAPs and autism spectrum disorder (ASD) among children at age eight (77). Exposure to ambient metals, PM, and volatile organic pollutants were estimated at the census-tract level of birth residence using the 1996 National Air Toxics Assessment annual-average model. This study found that prenatal exposure to air pollutants including quinoline (OR = 1.4, 95% CI = 1.0–2.2), and styrene (OR = 1.8, 95% CI = 1.0–3.1) were associated with elevated risk for autism at age eight after adjusting for important covariates (Table 2). Besides the marginally significant results, this study is also limited by several weaknesses. First, exposure misclassification is possible due to the lack of information on activity patterns. Second, the use of children with other developmental disorders as controls could have biased results towards the null because it is possible that children with developmental disorders in general are more exposed to pollution.

Volk et al., in their recent study investigating the effects of proximity to freeway on risks of autism, found that after controlling for demographics and maternal smoking status, multiple logistic regression models show that pregnant mothers living more than 309 m away from freeway have an 86% increased risk of having the child with autism (OR = 1.86 95% CI: 1.04–3.45). More importantly, this study also made an attempt to investigate time of exposure – which many studies for children we reviewed did not address – and found that exposure during third trimester is associated with 2.22 times the risk (OR = 2.22, 95% CI 1.16–4.42) (78). Since this study requires accurate information on residential information during different trimester of pregnancy, recall bias could be a potential limitation.

Becerra et al. conducted a case–control study investigating the effects of TRAP exposure during pregnancy on development of autistic disorder (AD) among children born in 1995–2006 in California. The prenatal exposures to air pollutants were measured using both the nearest air monitoring stations and a LUR model. The study indicated that 12–15% increases in odds of AD were associated with per inter quartile range increase for ozone (OR: 1.12, 95% CI: 1.06–1.19; per 11.54-ppb increase) and PM ≤ 2.5 μm (OR: 1.15; 95% CI: 1.06–1.24; per 4.68-μg/m^3^ increase) after adjusting for both maternal and perinatal characteristics (includes SES). Selection bias may likely exist in this study (79).

Another prospective cohort study was used to investigate the associations between long-term exposure to air pollution and newly diagnostic ASD among 49,073 children <3 years in Taiwan. The results indicated that risk of newly diagnostic ASD was increased with an increase in ozone exposure (Adjusted HR: 1.59, 95% CI: 1.42–1.78 per 10 ppb increase); CO (HR = 1.37, 95% CI 1.31–1.44); NO2 (HR = 4.43, 95% CI 3.33–5.90), and SO2 (HR = 1.18, 95% CI 1.09–1.28 per 1-ppb increase). Cohort study design, larger sample size and longer follow-up period are some strengths of the study. The main study limitation is potential residual confounding (80).

Roberts et al. also conducted a case–control study investigating the effect prenatal exposure to HAPs and ASD among children of participants in the Nurses’ Health Study II (325 cases, 22,101 controls) and found that the perinatal exposures to highest quintile of diesel (highest OR: 2.0, 95% CI: 1.0–4.0), lead (1.6, 95% CI: 1.1–2.3), Mn (1.5, 95% CI: 1.1–2.2), cadmium (1.5, 95% CI: 1.0–2.1), and overall metals (1.6, 95% CI: 1.1–2.4) were significantly associated with development of ASD compared the lowest quintile. Gender might modify the effects of HAPs on ASD. Despite large national sample size, this study is limited, such as inaccurate measurement of exposures and collection of unreliable prenatal residency information during or post pregnancy (81). Raz et al. also conducted a nested case–control study (245 cases and 1522 controls) to examine the association between maternal exposure to PM air pollution and ASD in the offspring of those participants in the Nurses’ Health Study II. Diagnoses of ASD were based on maternal report and exposure to PM_10_ and PM_2.5–10_was estimated with the validated spatiotemporal model. It was found that maternal exposure to PM_2.5_ during pregnancy was associated with greater odds of ASD in children. In addition to exposure misclassification due to lack of the exact date of residential change and activity pattern information, the participants as all nurses may limit the generalizability of this study (84).

Another case-control study (78) examined the effects of TRAP on autism (279 cases, 245 controls) among those enrolled in the Childhood Autism Risks from Genetics and the Environment (CHARGE) Study in California. The residential information was collected using questionnaire. TRAP exposure at residency was estimated using a line-source air quality dispersion model. This study found significant associations among cases living in highest quartile between TRAP exposure in first year of life (OR = 3.10, 95% CI: 1.76–5.57), all pregnancy (OR = 1.98, 95% CI: 1.20–3.31), first trimester (OR = 1.85, 95% CI: 1.11–3.08), second trimester (OR = 1.85, 95% CI: 1.11–3.08) and third trimester (OR = 2.10,95% CI: 1.27–3.51) and autism. This study is limited due to effect of confounding from relating factors, such as lifestyles, nutrition, other residential exposures, and proximity of physician diagnosis (82).

Similarly, Kalkbrenner et al. studied the effects of exposure to PM_10_ on autism spectrum disorder (autism). A total of 645 cases in North Carolina and 334 cases in the San Francisco Bay area were selected. Children in the control group were randomly selected from birth certificate based on counties and birth years of the selected cases. Geo-statistical interpolation method was used to assess individual exposure to PM_10_ during within 3-month periods of preconception through the first year of life. This study indicates exposure PM_10_ during the third trimester of pregnancy was associated with autism. The study has enough sample size and was able to control for maternal socioeconomic factors, while it is lack of the data for other pollutants, such as PM_2.5_ and NO_2_, which have been studied in other studies (83).

A population-based case–control (217 cases, 226 controls) study was conducted to investigate the risk of ASD due to prenatal and early childhood exposure to PM_2.5_ among children born between 2005 and 2009 in six counties in Southwestern Pennsylvania (85, 86). Exposure to PM_2.5_ was estimated using the LUR. This study found that prenatal exposure and postnatal exposure to PM_2.5_ are significantly associated with increased risk of ASD. The strengths of this study include having residential histories for PM_2.5_ exposure assessment and several windows of exposure from 3 months prior to conception through the second year of life to be considered (85). Additionally, based on the same study subjects, the investigators also examined the association between ASD and HAPs. The levels of 30 selected neurotoxicants at census tracts of residential address were assessed using the 2005 US EPA NATA data. This study reported that mothers living in areas with higher levels of styrene and chromium during pregnancy had a higher risk of ASD among their offspring. The limitations of this study include a semi-ecological design that assumed that all individuals at the same census tract had the same exposure levels of HAPs and lack of longitudinal data of HAPs (86). Guxens et al. used four European population-based birth/child cohorts to examine the effects of prenatal air pollution exposure on childhood autistic traits among general population (>8000 children). LUR models were applied to estimate nitrogen oxides and PM at birth residency. This collaborative study found that prenatal exposure to NO_2_ and PM was not associated with autistic traits at ages of 4–10 years old in children. This study consists of large sample size subjects with prospective and longitudinal study design, considering it as the most important strength (87).

##### Attention-Deficit Hyperactivity Disorder

There were also a few studies which have been conducted to examine the effects of air pollution on ADHD. Siddique et al. in Delhi, India investigated the relationship between vehicular air pollution and ADHD in children through a cross-sectional study and has found a dose–response relationship between PM_10_ exposure and risk of ADHD in children after controlling for age, SES, and BMI in a logistic regression model. Exposure to outdoor air pollution was determined by fixed site monitoring stations, and was adjusted for indoor levels measured by battery operated monitors. This study is limited by two major factors: (a) the cross-sectional design prevents causal inference, and (b) exposure based on fixed monitor stations are likely subjected to misclassification as they are not reflective of personal exposure. Despite weaknesses, these findings show some evidence that ambient air pollution may have adverse neurologic effects relevant to many neurological disorders, such as ADHD (73).

Newman et al. investigated the association between elemental carbon attributed to traffic (ECAT) and ADHD symptoms at 7 years of age in a birth cohort from the Cincinnati Childhood Allergy and Air Pollution Study (CCAAPS). ECTA exposure during the first year of life was estimated based on the data from air monitoring stations and LUR modeling. Significant association was observed between exposure to high ECAT and at-risk score for hyperactivity (1.7, 95% CI: 1.0–2.7) after controlling for sex, cigarette exposure during the first year of life and maternal education. In addition to it, significant increase in at risk scores for hyperactivity was associated with exposures to higher air pollutants among children born to mothers with higher education (1.7, 95% CI: 1.0–2.7) but the sample size was limited to children born to mothers with higher education attainment. The positive association could be explained as, higher maternal education could increase the school achievement expectations in mothers, whose reports might be higher for their children behavioral concerns. The selection of study participants was based on high risk for atopy, which may limit the actual results (74).

Gong et al. conducted a cohort study on the effects of pre and postnatal exposures to TRAP and its association with ASD and ADHD among 3426 twin children born in Stockholm in 1992–2000. The neurodevelopment outcomes of the children were screened via telephone interviews and air pollution exposure assessment were assessed using residence time-weighted concentrations of PM_10_ and NOx based on b dispersion models. However, this study did not support an association between prenatal or postnatal air pollution and ASD or ADHD. Several factors could have grounds discrepancy in the study results, such as presence of considerably low level of pollution and effects of other unselected confounders, such as maternal smoking status, including SES variables might have led to inconsistent associations. The major study limitation includes probable inconsistency in neurodevelopment outcomes data obtained from those participating in CATSS and general population (75).

Overall, studies on the effects of pollution in young children have – similar to those for adults – lacked the adjustment of important confounders, such as community factors, genetics underpinnings, and more importantly, the investigation of a critical window of exposure. While it is difficult to prove the importance of a prenatal critical window of exposure due to the fact that diagnoses are often made long after birth, it is still informative to study the time during which exposure has the strongest association with neurologic health outcomes. Although several studies we reviewed have made some attempt to reveal critical period of exposure by measuring exposure at specific time during pregnancy (e.g., third trimester), it remains inconclusive. Furthermore, studies that examined neurocognitive functions used various tests, making comparison between studies difficult. There is a clear need to unify these methods in air pollution studies.

Although the number of epidemiological studies is still small and varies in methodology, evidence from cross-sectional studies, case–control studies, and cohort studies suggests that exposure to ambient or traffic air pollution could potentially cause adverse health effects on neurobehavioral functions in children. Especially, although the relationships between ambient air pollution and neurodevelopmental disorders, such as ADHD and autism, have been increasingly studied, evidence is still limited and inconsistent. Future research on this field may be needed as the increasing trends of these diseases over the past years.

## Discussion

As mentioned earlier, the fact that neurological effects ranked second out of the seventeen health effects of ambient air pollution suggests that ambient air pollution plays a critical role in neurologic health. Thus, the study of neurological effects of ambient air pollution is an important emerging field of environmental epidemiology. Our systematic review suggests that there is evidence linking ambient air pollution with adverse neurological effects in both adults and children. Overall, consistency was demonstrated across studies.

Given the relative infancy of this research area, most of the publications occurred over the last 5 years. A critical evaluation of the evidence is therefore timely, appropriate and necessary. The reported associations between air pollution and neurological effects will be discussed regarding the following aspects: measurement of exposures, confounding and outcomes, and biases. This evaluation is intended to help guide developing future studies in this field.

### Measurement of Exposure

As direct associations between air pollution and adverse neurological effects need to be further established, exposure assessment of air pollution is a crucial element for future study. The effect of ambient air pollution on the nervous system is most likely to be long term or chronic in nature. With current technology typically used in population-based studies, measurement of exposure to air pollution is most likely to represent an exposure during a specific window in a lifetime. Challenges in the measurement of air pollution exposure include duration of exposure, residential and occupational stability, personal time-activity patterns, and temporal and spatial variation of air pollution. Many studies reviewed above used a binary exposure measurement, i.e., high polluted area vs. low polluted area, annual concentrations of air pollutants from surrounding monitors or distance to traffic ways as an estimate of air pollution. These exposure measurements can be problematic and misclassification of exposure is very likely. For this reason, it is crucial to develop more accurate methods to measure chronic exposure to air pollution in this field. In children, a few studies that we reviewed tried to remedy the issue by using a biomarker, such as PAH–DNA adduct level in cord blood (69) and personal monitoring (71).

#### Biomonitoring

Biomarkers, mainly DNA adducts measured by ^32^P-postlabelling (94) and PAH–DNA adducts level tested by enzyme-linked immunosorbent assays (95), are valuable to measure chronic exposure of air pollution. Studies suggest that DNA adducts level is highly associated with ambient air pollution (96–98). Only two of the many studies we reviewed used this method for exposure estimation (66, 71). However, even with these biomarkers, research still faces the difficulty of distinguishing the sources of exposure, which may also include exposures, such as cigarette smoking. For example, if a subject has high level of PAH–DNA adduct, it is possible to tell whether the person has high chronic exposure to the chemicals; however, it is not possible to discern whether the person is exposed through ingestion, inhalation or other routes of exposure. In addition, measuring DNA adducts level requires a blood sample, which are more invasive, time-consuming and expensive than many population-based studies can accommodate. Another limitation regarding this method of exposure measurement is the fact that peripheral markers may not reflect the environment in the CNS. More importantly, since events in the CNS vary considerably by brain region, peripheral markers may not be very relevant in some cases.

#### Personal Monitoring

Personal monitors are often considered as the most accurate estimate of individual air pollution exposure. Among the studies we reviewed, only two used this method (64, 69). For most studies this type of measurement is not feasible due to high costs, a requirement of prospective cohort study design, and necessary length of measurement time. Nevertheless, studies in children may be promising given the flexibility in length of study time in children, particularly if examining a specific time window, such as prenatal period, which is a finite time period. Personal monitoring may provide an estimate of exposure less prone to misclassification than other methods of measuring air pollution exposure in children when a sufficient number of measures are taken in a specific window (69, 99, 100). However, this method is very difficult to use in the adult population to measure life cumulative or chronic exposure to air pollution because the application of the method requires prospective study design, long length of follow-up, and a large sample size. Regardless of feasibility, given the lack of availability of reliable tools for measurement of exposure to air pollution, personal monitoring currently yields the best estimation of exposure and therefore should be considered as the current gold standard.

#### Exposure Assessment by Modeling

In this context, geographic information system techniques, such as LUR and other geo-statistical models, may complement personal monitoring and biomonitoring methods. In LUR model, various GIS parameters, such as traffic density, population density, elevation, and land use, are used to predict the small-scale spatial variation of pollutants (101, 102). Therefore, the model can be used to estimate concentrations at home locations based on the spatially refined independent variable datasets (103). This model is, however, known to capture spatial variability instead of temporal variability. The LUR model has mainly been used to examine long-term (e.g., over years or lifetime) exposure to air pollution (104, 105). Some studies we reviewed used the method of LUR models in the exposure assessment (35, 36, 53, 58). One of the limitations that these studies faced was estimating air pollution levels on current residential address, which ignores historic exposures. People are mobile and are not confined in the place where exposure was assessed leading to potential misclassification of exposure. In order to reduce the potential for exposure misclassification, efforts should be made to include information on individual residential history and duration of stay in LUR models. Subjects’ activity pattern is also important and should be controlled for in statistical analysis.

#### Critical Window of Exposure

The timing of exposure to a neurotoxic agent is one of the most important factors determining the effects produced by a neurotoxic agent, particularly given the potential for accumulation over the life course. However, it is also possible that air pollution may affect the nervous system in other ways that can influence development of disease. Depending on the health outcome under study, there may be a limited window in which air pollution can have adverse effects or has stronger effects on the nervous system than other times in life, i.e., a critical or sensitive period (106). For example, prenatal exposures to different pollutants have been consistently found to have profound effects on postnatal life in infants (64, 69, 71). Consequently, identifying a critical window of exposure during prenatal period is essential in determining whether there is a causal relationship between air pollution and the nervous system. Furthermore, the time window is important with respect to developing appropriate preventive strategies for reducing adverse neurological health effects of air pollution. However, research on this specific area regarding the neurological effects of air pollution is still limited, as suggested by the fact very few of the studies reviewed addressed this issue. In children, neurotoxic effects of prenatal and postnatal air pollution exposure are far less clear than other points in their life. Similarly, none of the research we reviewed has examined the susceptibility window of neurotoxicity of air pollution in adult populations.

While a critical window of exposure is important in understanding the effects of air pollution and to develop effective interventions, it is still important to recognize that it is difficult to attribute negative effects to only the critical window period. The problem lies in the fact that most health endpoints are diagnosed after birth, making it very difficult to rule early life effect and conclude that prenatal exposure is more relevant. Moreover, there is evidence that suggests acute effects of air pollution on different health outcomes, suggesting that acute effects might also be important for neurological endpoints (107–109).

### Confounding

The nervous system is sensitive to multiple environmental exposures. A number of environmental neurotoxicants, such as lead, mercury, polychlorinated biphenyls, pesticides, and ionizing radiation, have been well reported (110–113). Therefore, controlling for the confounding effects of these factors is necessary for an accurate estimation of the health effects of air pollution on the nervous system. However, for some environmental pollutants, such as lead, air pollution is also one of the primary sources of exposure (114). A complete adjustment of the effects of chemicals may result in underestimating the health effects of air pollution because they may be the important components of air pollution. Under this circumstance, it becomes more complex to control for the confounding effects of these chemicals. Furthermore, as metal pollutants are highly correlated in most cases, the adjustment of all others in order to find the effect of one pollutant makes the multi-pollutant model extremely complicated. It may lead to higher chances of finding false negative results, due to over-adjustment. Moreover, since individual pollutants have been found to be affecting neurological outcomes as discussed throughout the paper, multiplicative and additive effects are possible; none of the study we reviewed has examined these interactions.

Confounding due to lifestyle factors, such as smoking and alcohol drinking, may also be problematic, as these factors are also associated with health effects on the nervous system and may also be associated with air pollution exposure, given correlations with living in disadvantaged neighborhoods (115, 116). Several studies have consistently reported that smoking including active smoking and passive smoking can cause cognitive impairment in both adults and children (117–120). However, other studies found that alcohol consumption was beneficial to cognitive functions in adults (121–124). Thus, the potential confounding effects of these factors are required to be considered differently when examining the health effects on the nervous system.

Recognizing environmental justice issues in this literature also warrant attention in terms of the potential confounding effects of individual-level and area-level socioeconomic position on the relationship between environmental exposures and health outcomes. For example, Miranda et al. have found that poorer and minority neighborhoods are more likely to experience higher pollution levels. In addition, socioeconomic factors are also highly correlated with higher disease burden. Despite the important need to adjust for these confounders, very few of the studies we reviewed adjusted for or examined effect modification by socioeconomic position of individuals or their communities (92).

Given that many health endpoints have genetic underpinnings, there is emerging yet another important confounding factor: gene–environment interaction (93). This suggests that there could be susceptible subpopulations that need more attention than others. However, none of the studies we reviewed addressed this issue, indicating that future studies in this field need to pay closer attention to gene–environmental interaction in order to make better inference, and to identify susceptible populations for prompt intervention.

### Publication Biases

The studies reviewed above were all selected from publications in major journals being identified through electronic databases and manual searches. There is a possibility that studies with negative or null results are less likely to be published in major journals, especially in the relatively early stage of development of this topic (125, 126). Consequently, the results might point more toward a positive association. In addition, publications in non-English journals were not included in the review. Therefore, we cannot exclude the possibility that these studies could also impact the results of the review. However, given the recent interest in this topic, most studies are more likely to be at least presented at conferences and found in conference proceedings, of which we also searched.

## Conclusion

Despite being a new area of research, there is evidence of association between air pollution and neurological function in both adults and children. Air pollution continues to be important environmental and public health concerns worldwide. Since the literature on the association between the nervous system and ambient air pollution is relatively new, there is no doubt that more research is required to fully understand the relationships. Future studies might be designed to use more accurate exposure measurement, to target different health endpoints (e.g., endpoints measured by neuropsychological tests, neurodevelopmental disorders and neurodegenerative diseases), to pinpoint one specific toxicant of importance including gaseous air pollutants, and to emphasize the combined neurological effects of multiple air pollutants. Furthermore, identification of susceptible subpopulations is the priority in air pollution studies. Future studies should also be designed to investigate the potential effect modifiers including genetic factors of the neurotoxicity of air pollution. Identification of factors that determine susceptibility will provide better ways to characterize susceptible groups within the population and guide to develop better health policies to protect the population. Finally, positive results from this effort could potentially lead to revision of current limits for the primary national ambient air quality standards for criteria pollutants to be set low enough to protect the health of all susceptible groups within the population and consequently contributes to meeting the requirement of the U.S. Clean Air Act (127).

## Author Contributions

Ms. RB, Dr. XX, and Dr. SH are responsible for literature review and manuscript drafting.

## Conflict of Interest Statement

The authors declare that the research was conducted in the absence of any commercial or financial relationships that could be construed as a potential conflict of interest.
